# Dysfunction of spatacsin leads to axonal pathology in *SPG11*-linked hereditary spastic paraplegia

**DOI:** 10.1093/hmg/ddu200

**Published:** 2014-05-02

**Authors:** Francesc Pérez-Brangulí, Himanshu K. Mishra, Iryna Prots, Steven Havlicek, Zacharias Kohl, Domenica Saul, Christine Rummel, Jonatan Dorca-Arevalo, Martin Regensburger, Daniela Graef, Elisabeth Sock, Juan Blasi, Teja W. Groemer, Ursula Schlötzer-Schrehardt, Jürgen Winkler, Beate Winner

**Affiliations:** 1 IZKF Junior Research Group and BMBF Research Group Neuroscience, IZKF, Friedrich-Alexander-Universitaet Erlangen-Nuernberg, Glueckstrasse 6, Erlangen 91054, Germany; 2 Department of Molecular Neurology,; 3 Department of Psychiatry and; 4 Department of Ophthalmology, Friedrich-Alexander-Universitaet Erlangen-Nuernberg, Schwabachanlage 6, Erlangen 91054, Germany; 5 Department of Pathology and Experimental Therapeutics, Universitat de Barcelona (UB)-Campus Bellvitge, Feixa Llarga s/n, 08907 L'Hospitalet de Llobregat, Barcelona, Spain; 6 Institute of Biochemistry Emil-Fischer Zentrum, Friedrich-Alexander-Universitaet Erlangen-Nuernberg, Fahrstrasse 17, Erlangen 91054, Germany

## Abstract

Hereditary spastic paraplegias are a group of inherited motor neuron diseases characterized by progressive paraparesis and spasticity. Mutations in the spastic paraplegia gene *SPG11*, encoding spatacsin, cause an autosomal-recessive disease trait; however, the precise knowledge about the role of spatacsin in neurons is very limited. We for the first time analyzed the expression and function of spatacsin in human forebrain neurons derived from human pluripotent stem cells including lines from two *SPG11* patients and two controls. SPG11 patients'-derived neurons exhibited downregulation of specific axonal-related genes, decreased neurite complexity and accumulation of membranous bodies within axonal processes. Altogether, these data point towards axonal pathologies in human neurons with SPG11 mutations. To further corroborate spatacsin function, we investigated human pluripotent stem cell-derived neurons and mouse cortical neurons. In these cells, spatacsin was located in axons and dendrites. It colocalized with cytoskeletal and synaptic vesicle (SV) markers and was present in synaptosomes. Knockdown of spatacsin in mouse cortical neurons evidenced that the loss of function of spatacsin leads to axonal instability by downregulation of acetylated tubulin. Finally, time-lapse assays performed in SPG11 patients'-derived neurons and spatacsin-silenced mouse neurons highlighted a reduction in the anterograde vesicle trafficking indicative of impaired axonal transport. By employing SPG11 patient-derived forebrain neurons and mouse cortical neurons, this study provides the first evidence that *SPG11* is implicated in axonal maintenance and cargo trafficking. Understanding the cellular functions of spatacsin will allow deciphering mechanisms of motor cortex dysfunction in autosomal-recessive hereditary spastic paraplegia.

## INTRODUCTION

Hereditary spastic paraplegias (HSPs) are a heterogeneous group of neurodegenerative disorders commonly characterized by the degeneration of long axons of the cortico-spinal tract and dorsal columns (1). At present, genetic mapping and exome sequencing have identified at least 71 HSP gene loci, designated *SPG 1-71*, and up to date 54 genes causing an autosomal-dominant, -recessive (AR-HSP) or X-linked inheritance (2). In particular, mutations in *SPG11* are the most frequent cause of AR-HSP. Mutations in *SPG11* were first described by screening patients with AR-HSP, thin corpus callosum (TCC) and cognitive impairment (3). Additional symptoms including pseudobulbar symptoms such as dysarthria and dysphagia, neuropathy and amyotrophy are also present in some patients with *SPG11* mutations (4,5).

So far, more than 102 *SPG11* mutations equally distributed within the gene have been described (available at ‘The Human Gene Mutation Database—HGMD’). *SPG11* encompasses 40 exons encoding for the 2443 amino acids protein, spatacsin (3). Very little is known about the structure and function of spatacsin. Together with spastizin (another AR-HSP protein, encoded by *SPG15*), spatacsin colocalized with vesicle, endosomal and endoplasmatic reticulum markers (6,7). Genomic screens as well as proteomic and biochemical assays demonstrated an association of spatacsin and spastizin with the adaptor protein 5 (AP5) (6,8,9). The knockdown of *spg11* in zebrafish compromised the outgrowth of spinal motor axons and suggested that spatacsin may be important for the formation of neuromuscular junctions during development (10).

In this study, we characterized for the first time neuronal cultures derived from human-induced pluripotent stem cells (hiPSCs) of SPG11 patients (SPG11-dNeurons), where we observed downregulation of specific axonal-related genes, decreased neurite complexity as well as accumulation of membranous deposits within the axonal processes.

To further characterize spatacsin function, we analyzed human pluripotent stem cell-derived neurons and mouse cortical neurons. Spatacsin colocalized with cytoskeletal structures and SV markers in neuronal cultures and synaptosomes. Specific silencing of spatacsin in mouse cortical neurons resulted in outgrowth defects, retrograde axonal retraction and downregulation of acetylated tubulin.

Functionally, time-lapse assays revealed that SV transport was severely impaired in axonal processes of SPG11-dNeurons and spatacsin-silenced mouse neurons.

In summary, human and mouse cortical neurons share similar expression patterns and protein localization of spatacsin. Moreover, assays performed in SPG11-dNeurons and spatacsin-silenced mouse cortical neurons delineated that spatacsin dysfunction leads to axonal pathology and vesicle trafficking defects. These results strengthen the relevance of spatacsin for axon maintenance due to its crucial role for transport mechanisms.

## RESULTS

### Spatacsin is present in human and mouse cortical projection neurons

**Figure 1.**
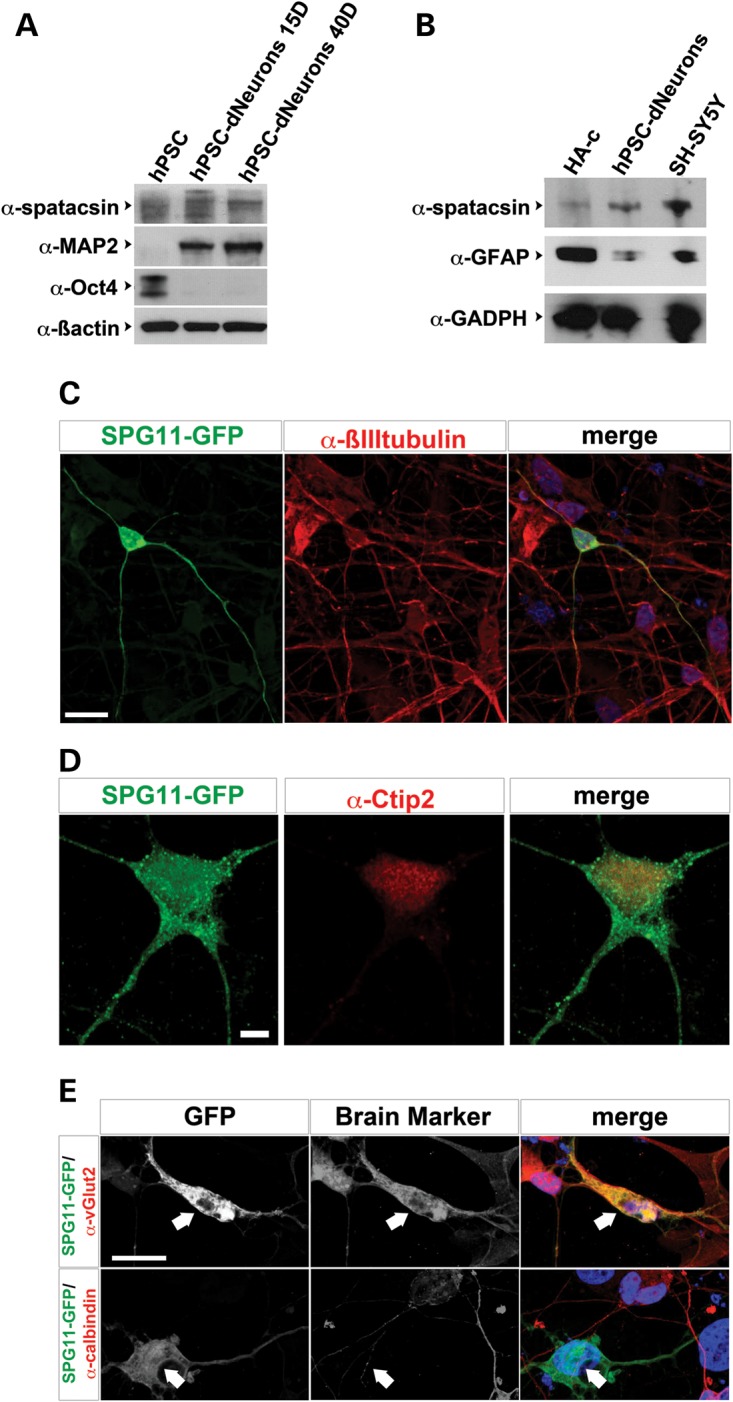
Characterization of spatacsin expression in human-derived neurons. (**A**) Blots of HUES6 (hPSC), HUES6-dNeurons cultured in neuronal differentiation media for 15 (hPSC-dNeurons 15D) or 40 days (hPSC-dNeurons 40D). Blots were probed with α-spatacsin. Oct4 and MAP2 were employed as stem cell and neuronal markers, respectively. β-Actin served as loading control. (**B**) Blots of homogenates from human astrocytes (HA-c), HUES6-dNeurons (hPSC-dNeurons) and SH-SY5Y cells were probed with α-spatacsin, α-GFAP and α-GADPH as loading control. (**C**) HUES6-dNeurons cultures transfected with SPG11::GFP (SPG11-GFP, green) were labeled with α-βIII-tubulin (red) and DAPI (blue). Scale bar = 20 µm. (**D**) HUES6-dNeurons cultures transfected with SPG11::GFP (green) were stained with α-Citp2 (red). Scale bar = 5 µm. (**E**) HUES6-dNeurons transfected with SPG11::GFP (SPG11-GFP; green) colabeled with antibodies against α-vGlut2 or α-calbindin (red; arrows). Scale bar = 20 µm.

Mutations in *SPG11* promote a progressive cortical degeneration (4). However, the expression pattern of spatacsin during the maturation of human neurons is unknown. First, we investigated the expression of spatacsin in human neurons derived either from human embryonic stem cells (HUES6-dNeurons, Fig. 1A) or from human-induced pluripotent stem cells (hiPSC-dNeurons; data not shown) and observed that spatacsin is expressed throughout neuronal differentiation. Furthermore, the detected spatacsin expression in HUES6-dNeurons was similar to blots using the human neuroblastoma cell line SH-SY5Y (Fig. 1B) (7), and higher compared with human astrocytes (HA-c; Fig. 1B). The specificity of the employed antibody was confirmed by isotype controls (Supplementary Material, Fig. S1A). Moreover, GFP-tagged-spatacsin (GFP-Spat) overexpressed in HEK293 cells was detectable with both GFP and spatacsin antibodies. Endogenous spatacsin expression was downregulated in HEK293 cells transfected with an siRNA against SPG11 (siSPG11; Supplementary Material, Fig. S1B and C).

We then expressed GFP under the regulation of the SPG11 promoter (SPG11::GFP) in HUES6-dNeuron cultures. GFP^+^ cells coexpressed with neuronal markers βIII-tubulin, the cortical marker Citp2 and the projection neuron marker vGlut2, but not with the neuronal marker calbindin (Fig. 1C–E).

### Spatacsin is expressed in embryonic and adult mouse neurons

The expression of spatacsin was further investigated by comparing mouse cortical neurons transfected with constructs containing a SPG11 promoter (SPG11::GFP) or a CMV promoter (CMV::GFP; Supplementary Material, Fig. S2A and B). We observed a preference of spatacsin for neurons, as >90% of SPG11::GFP-positive cells colabeled for the neuronal marker MAP2 compared with only 40% MAP2^+^ cells of the CMV::GFP population (Supplementary Material, Fig. S2B and C). A detailed phenotypic analysis of the SPG11::GFP transfected murine cultures revealed that the GFP expression was detected in vGlut2^+^ projection neurons and in vGAT^+^ interneurons, but never overlapped with the glial marker GFAP (Supplementary Material, Fig. S2D). Using a quantitative approach, we detected higher expression levels of spatacsin in projection neurons (134.8±6 fluorescent units per cell) than interneurons (79±4.4 fluorescent units per cell).

Our results in neurons derived from human pluripotent stem cell (hPSC) cultures evidenced spatacsin expression during the neuronal differentiation process. Thus, we decided to test the spatial expression of spatacsin during mouse brain development and in adulthood. Our blots showed spatacsin expression in all brain areas analyzed from embryonic (E18) and adult mice (P150) (Supplementary Material, Fig. S3A). Furthermore, immunohistochemistry of the adult mouse brain employing the spatacsin antibody (Supplementary Material, Fig. S4) confirmed that spatacsin was expressed at low levels in NeuN^+^ neurons from cortical Layers II to VI and at high levels in few neurons found in cortical Layers III and V (Supplementary Material, Fig. S3B and C). In mouse brains, we did not find any GFAP^+^ glial cell colabeling with spatacsin (Supplementary Material, Fig. S3D). These data are in accordance with previous findings reported by Murmu and collaborators (7). Altogether, this indicates a strong similarity in the expression of spatacsin in human and mouse neurons.

### Spatacsin distribution in axons and dendrites in cortical neurons

**Figure 2.**
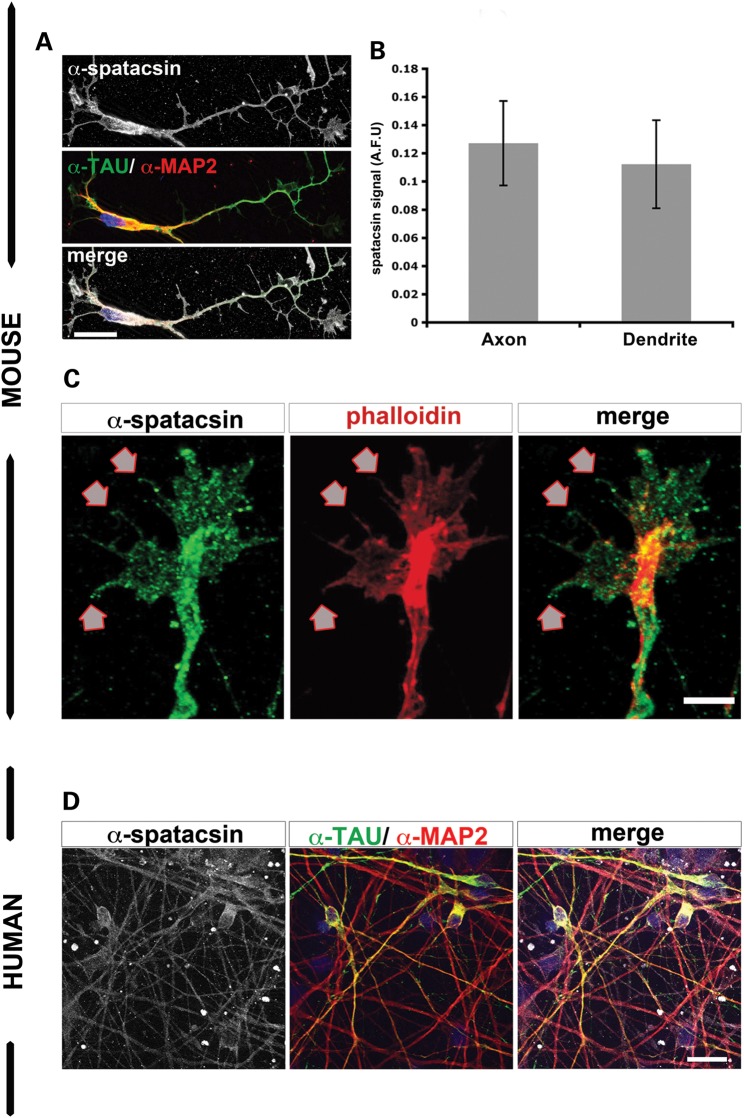
Spatacsin was present in the most distal tips of neurites of human pluripotent stem cell-derived neurons and mouse cortical neurons. (**A**) Mouse cortical neurons showed spatacsin expression (gray) together with the axonal marker α-TAU (green) and the dendritic marker α-MAP2 (red). Scale bar = 20 µm. (**B**) Graph for spatacsin expression as arbitrary fluorescent units (AFU) in axons (TAU^+^ neurites) and dendrites (MAP2^+^ neurites) of mouse cortical neurons; data represented as mean ± SD (*P* > 0.05); *n* ≥ 50 neurons per experimental condition were evaluated. (**C**) Spatacsin was observed in filopodia and membrane protusions (arrows) of growth cones of mouse cortical neurons. Scale bar = 5 µm. (**D**) Immunofluorescence analysis of HUES6-dNeuron cultures showed α-spatacsin (gray) overlapped with α-MAP2 (red) and α-TAU (green) markers. Scale bar = 50 µm.

Next, we investigated the specific subcellular localization of spatacsin within neurons. In mouse cortical neurons, we observed spatacsin expression in axonal (TAU) and dendritic (MAP2) processes (Fig. 2A) with no preference of spatacsin localization to either compartment (Fig. 2B). Furthermore, spatacsin revealed a cytosolic, punctuated expression pattern even in the most distal tips of filopodias and membrane protusions (Fig. 2A and C). Similarly, a cytosolic punctuated pattern of spatacsin expression was detected in HUES6-dNeuron cultures (Fig. 2D).

### Human neurons derived from SPG11 patients' iPSCs show reduced axonal plasticity

**Figure 3.**
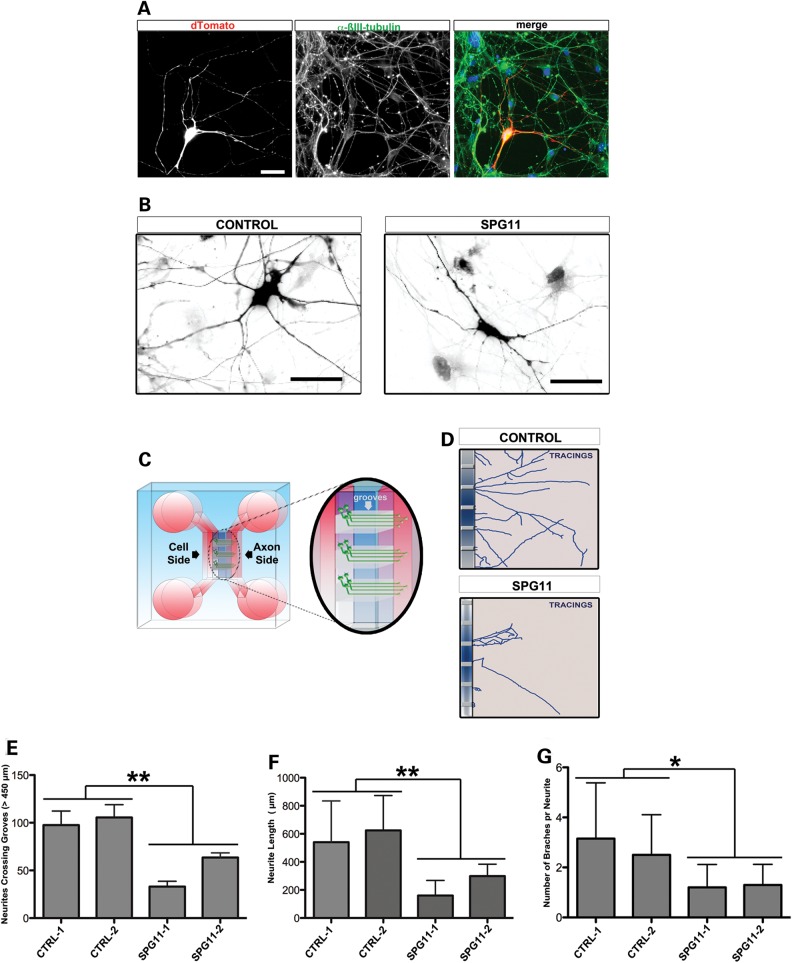
Significant reduction in axonal complexity of hiPSC-dNeurons from SPG11 patients. (**A**) Representative figure of hiPSC-dNeuron cultures transfected with pEF1-dTomato. The cells analyzed were coexpressing dTomato and the neuronal marker βIII-tubulin. Scale bar = 20 µm. (**B**) Neuronal cells from control and SPG11 patient, transfected with pEF1-dTomato, showing a marked decrease in neurite complexity. Scale bars = 50 µm. (**C**) Schematic representation of the microfluidic chamber showing the cell soma side and axonal side; inset shows the grooves in the chamber along which axons unidirectionally pass through. (**D**) Tracing of control and SPG11 neurites reaching the axonal side. Neurites in SPG11-dNeurons are shorter and have less branches compared with controls. (**E–G**) Graphs representing (E) the reduced number of neurites crossing the grooves, (F) the reduced total neurite length (µm) and (G) the reduced number of branching points in SPG11 compared with controls. Data are represented as mean ± SD (*P < 0.05 and **P ≤ 0.005); *n* ≥ 20 axonal processes per each cell line; two lines from CTRL-1 and CTRL-2 and two lines from SPG11-1 and SPG11-2 were employed.

We generated and characterized neuronal cultures derived from human-induced pluripotent stem cells (hiPSCs) of two SPG11 patients and two control fibroblast lines (Supplementary Material, Table S1) as described previously (11). All hiPSC lines used in this study were able to generate neuronal cultures with comparable percentages of neurons (∼70%) and glial cells (∼30%). We then analyzed the neuronal morphology by transfecting hiPSC-derived neurons with a pEF-1-dTomato construct to visualize single neurons within a dense culture as previously reported (11,12). We identified profound reductions in the neuritic complexity in neurons derived from SPG11 patients compared with controls (Fig. 3A and B). Due to the multidirectional growth of axonal processes (Fig. 3B), we plated the neuronal cultures in microfluidic chambers, which directed axonal processes to grow parallel and unidirectional along the grooves (Fig. 3C). This allowed us to measure subtle differences in neuronal architecture between SPG11 patient and control cells (Fig. 3D). Importantly, only 50% of the SPG11 axonal processes reached the axonal side of the chamber compared with controls (Fig. 3E) indicating a severely impaired outgrowth of SPG11 axonal processes. Moreover, in SPG11 patient-derived neurons, the axon length and the number of branching points per axon were significantly reduced (Fig. 3F and G). These analyses revealed for the first time that both neurite length and complexity are severely compromised in human neurons carrying SPG11 mutations.

### Spatacsin knockdown disrupts outgrowth in mouse cortical neurons

**Figure 4.**
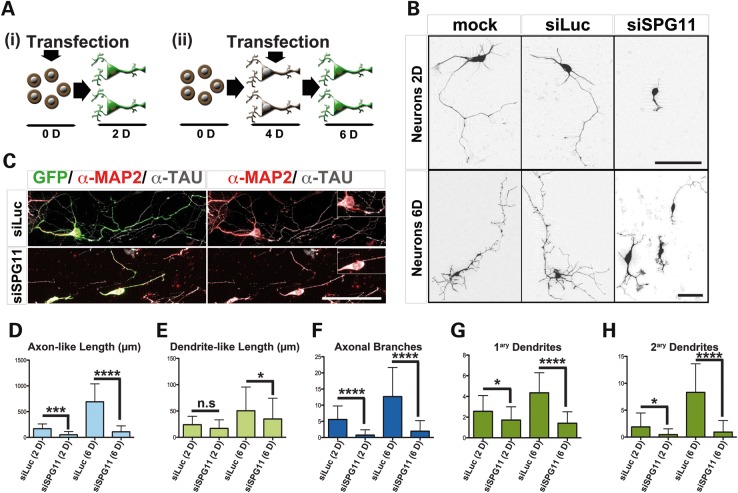
Knockdown of spatacsin impaired neurite outgrowth. (**A**) Scheme showing the two experimental strategies employed on mouse cortical neurons: (i) Dissociated mouse cortical neurons were transfected with siRNA together with GFP prior to seeding (Day 0) and then cultured for 2 days (Day 2). (ii) Neurons were transfected after 4 days in culture (Day 4). After transfection, cells were maintained for two additional days in culture (Day 6). (**B**) Photographs of mouse cortical neurons transfected with GFP (MOCK) only or together with either siLuc or siSPG11. The panel included examples of transfected neurons kept in culture for Day 2 (Neurons 2D, experiment i) and Day 6 (Neurons 6D, experiment ii). Scale bars = 50 µm. (**C**) Cortical neurons transfected with GFP (green) together with either siLuc or siSPG11 were colabeled with α-MAP2 (red) and α-TAU (gray). Scale bar = 50 µm. Note the abnormal MAP2-TAU overlap (insets) observed in siSPG11^+^ neurons in contrast to siLuc^+^ neurons. (**D–H**) Diagrams showing axonal length (D), dendrite length (E), number of branches per axon (F), number of dendrites per neuron (G) and the total number of secondary dendrites (H) in neurons transfected with siLuc or siSPG11 and cultured for Day 2 or 6. Measurements are presented as mean ± SD (*P < 0.05, ***P < 0.001 and ****P < 0.0001); *n* = 50–100 neurons per experimental condition.

Structural hallmarks of HSP caused by mutations in *SPG11* are the degeneration of cortico-spinal and transcallosal projections (13). Since the SPG11-derived human neurons exhibited a significant decrement in the complexity of the axonal processes, we hypothesized that spatacsin might play a crucial role in the maintenance and/or growth of axons. To analyze if loss of spatacsin is sufficient to generate these neurite phenotypes, we investigated neurite outgrowth in spatacsin-silenced mouse cortical neurons (Supplementary Material, Figs S1C and S5). In siSPG11^+^ mouse cortical neurons, we observed a marked reduction of neurite complexity at Day 2 compared with MOCK and siLuc transfected neurons (Fig. 4A, part i, B and D–H), paralleling our findings in SPG11 patient-derived neurons. It is well known that in mouse cortical cultures, the newly generated neurites specify between axon and dendrites within the first 2 days in culture (14–16). In accordance, we designed a second set of transfections in mouse cortical neurons at a time point when axons and dendrites had been specified (Fig. 4A part ii). Neurons transfected with siSPG11 at Day 4 showed significantly reduced length and branching complexity in both axon- and dendrite-like processes compared to Day 2 neurons (Fig. 4B and D–H). Moreover, we detected no significant differences in cleaved Caspase3^+^ cells between siSPG11 and siLuc neurons (data not shown). This indicated that the reduced bioavailability of spatacsin did not induce cell death in 6-day-old neurons. Noteworthy, all transfected cells evaluated in these assays stained positive for the neuronal markers βIII-tubulin (data not shown), TAU and MAP2 (Fig. 4C). By comparing the knockdown experiments after Days 2 and 6 (Fig. 4A and B), it is remarkable that the length of axon-like processes in siSPG11 neurons was shorter at Day 6 than control neurons at Day 2 (Fig. 4D and F). In accordance, MAP2^+^ and TAU^+^ processes at Day 6 were not fully specified in siSPG11 neurons compared with siLuc neurons (Fig. 4C).

**Figure 5.**
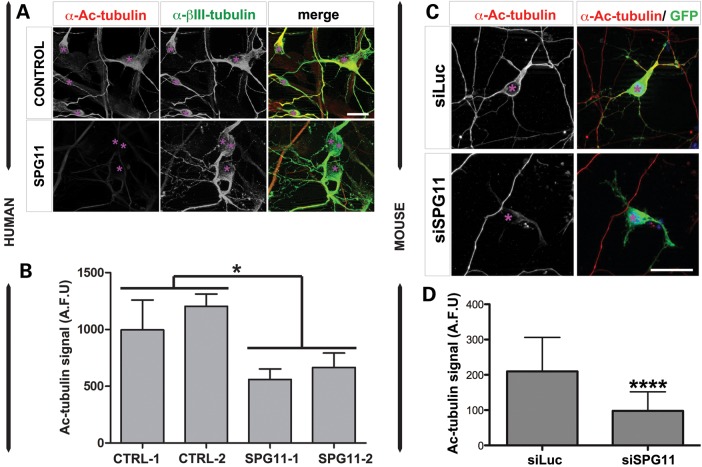
Reduction of acetyl-tubulin in hiPSC-dNeurons from SPG11 patients and spatacsin-silenced mouse neurons. (**A**) Cultures of hiPSC-dNeurons of SPG11 patients (SPG11) and healthy subjects (CONTROL) were colabeled with acetyl-tubulin (α-Ac-tubulin, red) and βIII-tubulin (α-βIII-tubulin, green). Scale bar = 10 µm. (**B**) Graph for acetylated tubulin signal (Ac-tubulin) in cultures of hiPSC-dNeurons of two controls (CTRL-1 and CTRL-2) and two SPG11 patients (SPG11-1 and SPG11-2). Levels of acetylated tubulin signal were significantly decreased in hiPSC-dNeurons of SPG11 patients compared with controls. Data were expressed as arbitrary fluorescent units (AFU), and represented as mean ± SD (*P < 0.05); *n* ≥ 100 neurons per experimental condition were evaluated. (**C**) Mouse cortical neurons transfected with pCMV-GFP (GFP, green) and either siLuc or siSPG11. Neurons were stained with α-acetyl-tubulin (red, α-Ac-tubulin). Scale bar = 20 µm. (**D**) Graph for acetylated tubulin signal (Ac-tubulin) in siLuc and siSPG11-transfected mouse cortical neurons. Levels of Ac-tubulin signal were significantly decreased following knockdown of spatacsin. Data were expressed as arbitrary fluorescent units (AFU) and represented as mean ± SD (***P < 0.001); *n* ≥ 50 neurons per experimental condition were evaluated.

Next, we asked if the reduced neurite outgrowth and retraction of processes reflect alterations in the stability of the microtubules, and hence we investigated the level of acetylated tubulin, a posttranslational marker of stabilized tubulin. Interestingly, we observed a significant reduction of acetylated tubulin signal in neurons derived from SPG11 patients (Fig. 5A and B), as well as in spatacsin-silenced mouse cortical neurons (Fig. 5C and D), indicating that the dysfunction of spatacsin may interfere with the stabilization of microtubules.

### Spatacsin associates with synaptic markers in human and mouse neurons and is essential for axonal transport

**Figure 6.**
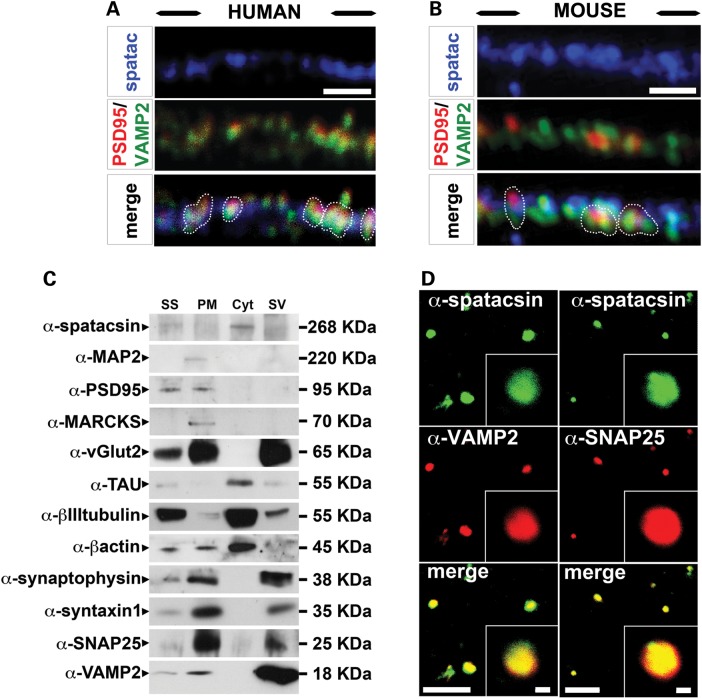
Characterization of spatacsin expression in synapses. (**A** and **B**) Spatacsin (blue) partially overlapped (indicated in white dotted lines) with the presynaptic marker VAMP2 (green) and the postsynaptic marker PSD95 (red) in HUES6-dNeurons (A) and in mouse cortical cultures (B). Scale bars = 1 µm. (**C**) Blots of samples from mouse synaptosome (SS) and the following synaptosomal factions: synaptosomal plasmatic membrane (PM), synaptosomal cytosol (Cyt) and synaptic vesicles (SV). Blots were probed with α-spatacsin, the postsynaptic markers α-PSD95 and α-MARCKS, the presynaptic markers α-SNAP25 and α-syntaxin1, the vesicle markers α-vGlut2, α-synaptophysin, and α-VAMP2; and the cytoskeleton markers α-MAP2, α-TAU, α-β-actin and a-βIII-tubulin. Spatacsin was predominantly in the Cyt fraction. (**D**) Purified mouse synaptosomes probed with α-spatacsin (green) together with either α-VAMP2 or α-SNAP25 (red). Scale bars = 40 and 1 µm in overviews and insets, respectively.

Genetic approaches in zebrafish revealed that the disruption of *spg11* promotes motor defects in embryos due to a significant reduction of neuromuscular junctions, the specialized synapses of the motor endplate (10,17). Both in HUES6-dNeuron and mouse cortical neuron cultures spatacsin partially overlapped with pre- (VAMP2) and postsynaptic (PSD95) markers (Fig. 6A and B). In the light of these observations, we sought to determine if spatacsin is present in synapses by taking a biochemical approach to investigate the adult mouse brain. We thus isolated synaptosome fractions from mouse forebrains and subsequently, synaptosome preparations (SS) were further fractionated into synaptosomal plasmatic membrane (PM), cytosolic (Cyt) and SV fractions. Spatacsin was predominantly detected in the Cyt fraction (Fig. 6C). Interestingly, besides spatacsin, the cytoskeletal markers TAU, βIII-tubulin and β-actin were also found in the Cyt fraction (Fig. 6C). However, when we analyzed whole synaptosome preparations using confocal microscopy, we observed that the spatacsin signal overlapped with the vesicle marker VAMP2 and the presynaptic marker SNAP25 (Fig. 6D), pointing towards distinct roles of spatacsin within synapses.

**Figure 7.**
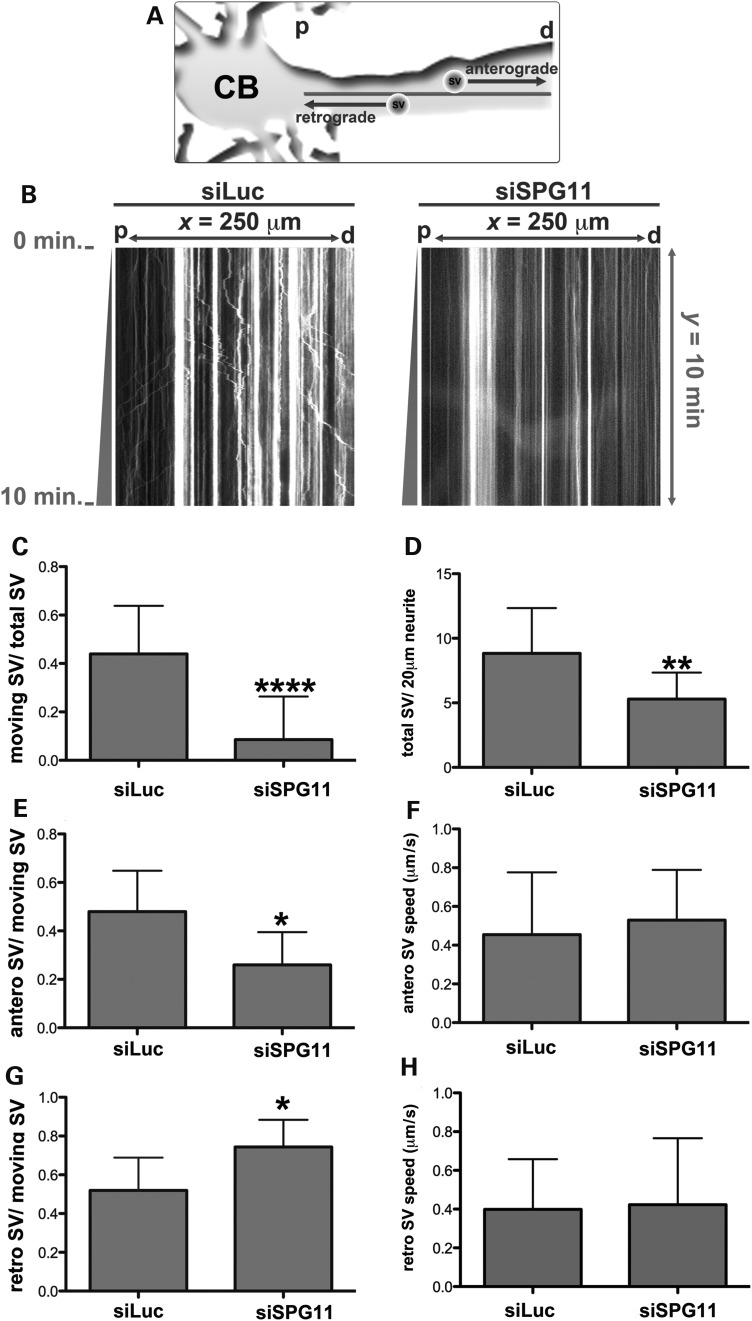
Knockdown of spatacsin disrupts SV transport. (**A**) Illustration of time-lapse monitoring of SV transport. SVs were visualized in neurons by expressing synaptophysin-mCherry. The neuronal cell body (CB) was used as a reference to regionalize proximal (p) and distal (d) neurite regions, and thereby distinguish the directions of anterograde and retrograde transports. (**B**) Kymographs representing SV transport in neurites transfected with synaptophysin-mCherry together with siLuc or siSPG11. The *x*-axis represents the neurite length (*x* = 250 µm) from proximal (p) to distal (d) areas. The *y*-axis indicates time-lapse duration in min (*y* = 10 min). Vertical lines exemplified stationary SVs (*x* < 5 µm), trajectories with × ≥ 5 µm considered moving SVs. Movements toward ‘p’ or ‘d’ revealed retrograde or anterograde transport, respectively. (**C–H**) Graphs indicate a significant decrease in the number of moving SVs and the average SV speed. All data were represented as mean ± SD; *n* ≥ 20 axons per experimental condition. (C) The ratio of total moving SVs in relation to the total number of SVs (****P < 0.0001). (D) The ratio of total SVs (total SV) per each 20 µm of neurite (**P ≤ 0.005). (E) The ratio between SVs moving anterogradely (antero SV) in relation to the total number of moving SVs (*P < 0.05). (F) The average speed of SVs moving anterogradely (antero SV speed in µm/s) (*P* > 0.05). (G) The ratio between SVs moving retrogradely (retro SV) in relation to the total number of moving SVs (*P < 0.05). (H) The average speed of SVs moving retrogradely (retro SV speed in µm/s; *P* > 0.05).

The expression of spatacsin in mouse cortical synapses and axonal outgrowth defects might imply a role of spatacsin in axonal transport mechanisms. Thus, we decided to investigate if disruption of spatacsin alters axonal transport by measuring anterograde and retrograde traffic of SVs in mouse cortical neurons transfected with siSPG11 and the fluorescent synaptic marker synaptophysin-mCherry (Fig. 7A). Interestingly, we found 40% of the evaluated siSPG11^+^ neurites lacked SV movements. The remaining siSPG11^+^ neurites had a significant reduction of mobile SV and moreover, the average number of SV per neurite length was decreased in siSPG11^+^ cortical neurons compared with siLuc^+^ neurites (Fig. 7B–D; Supplementary Material, Videos S1 and S2). A comprehensive evaluation of axonal transport directions revealed that anterograde traffic was proportionally reduced (Fig. 7E and G). However, the average speed of SV in siLuc^+^ and siSPG11^+^neurites was not different (Fig. 7F and H). Altogether, these findings suggest that loss of spatacsin causes a severe defect in the initiation and the direction of SV movement in mouse cortical neurons.

**Figure 8.**
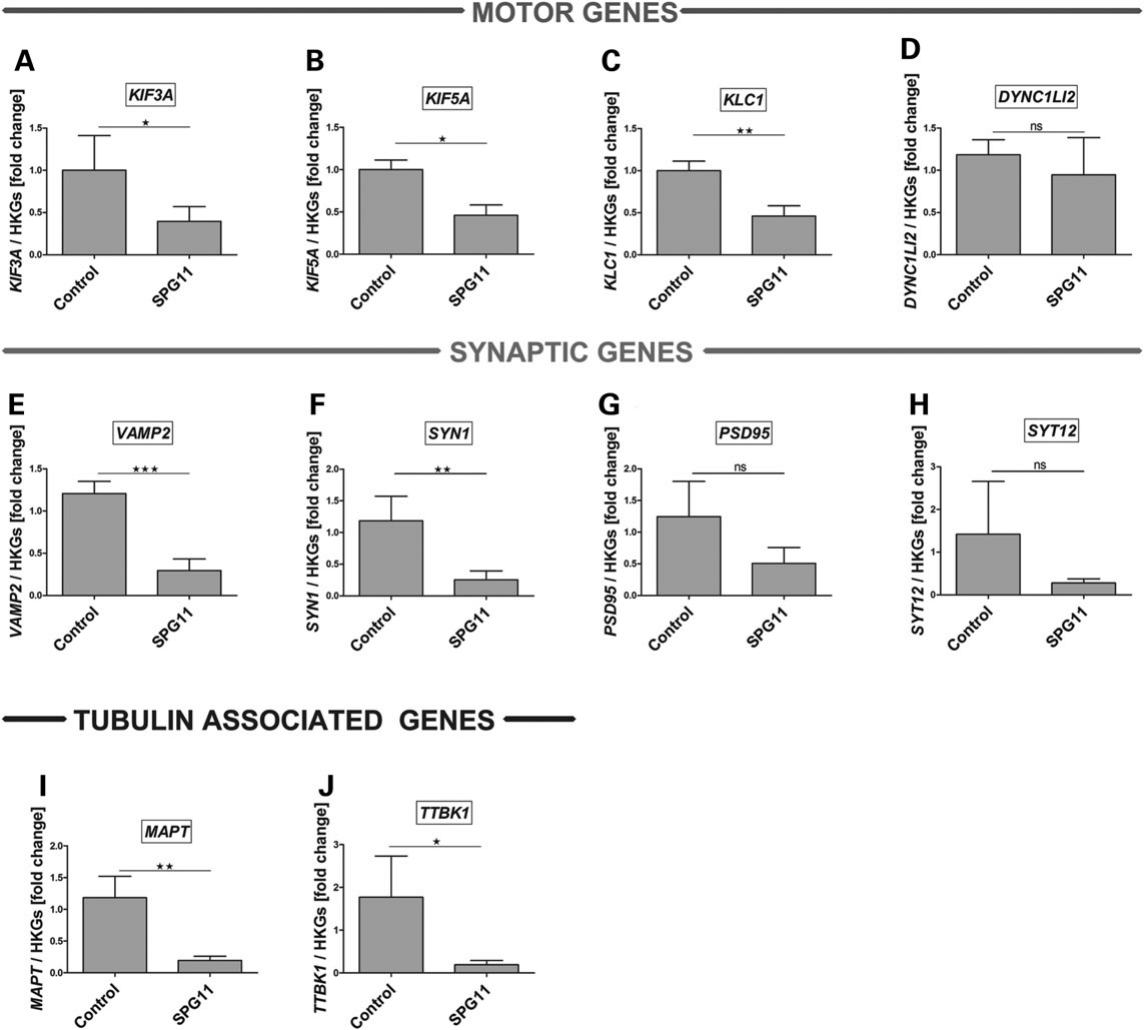
Expression analysis of transport-related genes in hiPSCs-derived neurons. RT-PCR analysis of genes in SPG11 patient-derived neurons revealed a significant reduction in the mRNA expression of kinesin-related genes, *KIF3A* (**A**), *KIF5A* (**B**) and *KLC1* (**C**), but no difference in the expression level of DYNC1LI2 (**D**) compared with control. Similarly, expression of synaptic genes, *VAMP2* (**E**), *SYN1* (**F**), but not postsynaptic density protein 95 (*PSD95)* (**G**) and synaptotagmin 12 (*SYT12*) (**H**) are strongly reduced in patient neurons. A strong downregulation of the cytoskeletal tubulin-associated genes, *MAPTAU* (**I**) and TAU tubulin kinase 1 (*TTBK1*) (**J**) further suggested dysregulation of transport activity in the patient neurons. Plotted are means of each line performed in triplicates, from two independent experiments. Data shown as mean ± SD. mRNA levels were normalized against two housekeeping genes (HKGs = *GAPDH* and *β2M*).

Our results from the mouse model revealed that spatacsin disruption intervenes axonal stability and SV transport. To corroborate these findings in human SPG11 pathology, we first investigated the mRNA expression analysis of the genes encoding important proteins involved in the transport machinery using real-time PCR (RT-PCR) assays on RNA isolated from hPSC-derived neurons. Indeed, in SPG11-dNeurons, we were able to detect a significant reduction of the expression of kinesin motor proteins, including *KIF3A*, *KIF5A* and kinesin light chain 1 (*KLC1*) which are involved in anterograde transport processes (Fig. 8A–C). In contrast, we did not observe significant expression differences in the retrograde motor protein, dynein cytoplasmic1 light intermediate chain 2 (DYNC1LI2). Next, we checked for presynaptic markers including vesicle-associated membrane protein 2 (*VAMP2)* and synapsin 1 (*SYN1*) (Fig. 8E–F) and cytoskeleton-associated genes including, microtubule associated protein tau (*MAPTAU*) and TAU tubulin kinase 1 (TTBK1) (Fig. 8I and J). Indeed, we could detect a significant downregulation of these associated genes. More striking was the fact that neuronal markers like postsynaptic density protein 95 (*PSD95*) and synaptotagmin 12 (SYT12) (Fig. 8G and H) were not altered thereby implying that the dysfunction of *SPG11* only disrupts the expression of specific subsets of neuron-related genes. These results implicated that loss of spatacsin might have a detrimental effect on the transport activity of human neurons.

**Figure 9.**
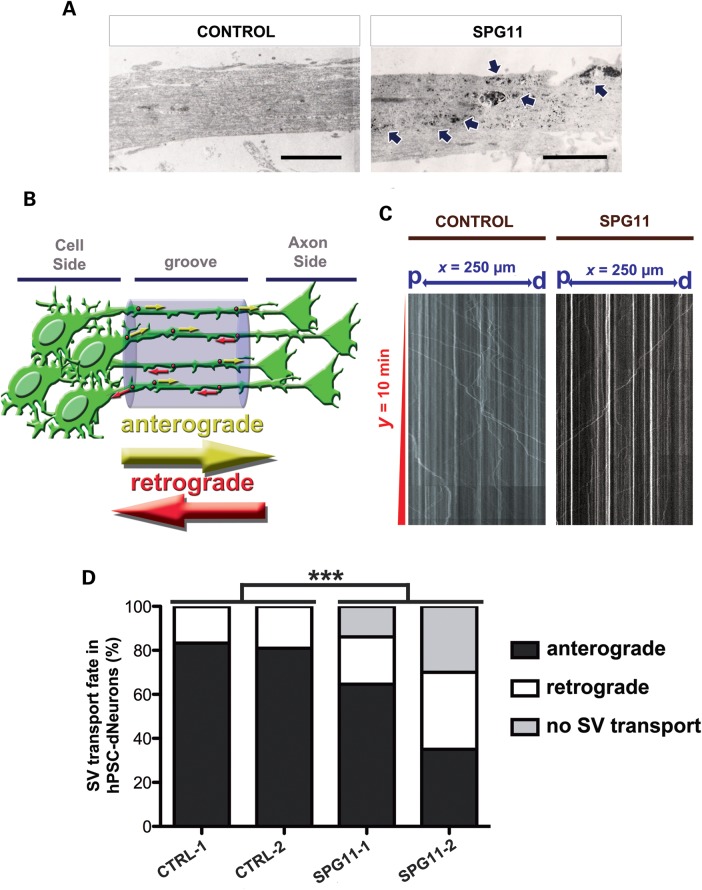
Human neurons derived from SPG11 iPSCs accumulate membrane-like deposits within neurites and compromise anterograde transport of SVs. (**A**) Ultrastructural analysis of the axonal processes of hiPSC-dNeurons from control (CTRL-1) and SPG11 patient (SPG11-1). Arrows indicate the presence of membranous inclusions in SPG11 neurons. Scale bars = 2 µm. (**B**) Illustration of time-lapse monitoring for SV transport in synaptophysin-mCherry^+^ hiPSC-dNeurons grown in microfluidic chambers. SVs mowing towards either the axon or the cell side were considered as anterograde and retrograde transports, respectively. (**C**) Kymographs representing SV transport in synaptophysin-mCherry^+^ axonal processes of neurons derived from controls (CONTROL) and SPG11 (SPG11) hiPSCs . The *x*-axis represents the distance (*x* = 250 µm) between cell side (p) and axon (d) sides. The *y*-axis indicates time-lapse duration in min (*y* = 10 min). Vertical lines exemplified stationary SVs (*x* < 5 µm), trajectories with *x* ≥ 5 µm were considered moving SVs. Movements toward ‘p’ or ‘d’ revealed retrograde or anterograde transport, respectively. (**D**) Graphs indicated a significant difference in the transport fate of SPG11 neurons (SPG11-1 and SPG11-2) in comparison to controls (CTRL-1 and CTRL-2). All data were represented as mean ± SD; ***P < 0.005; *n* ≥ 20 axons per experimental condition.

Previously, a significant increase of membranous structures in murine spastizin knockout neurons and human fibroblast of SPG15 patients were described (18,19). This prompted us to examine the content of vesicle organelles in hiPSCs-derived neurons of HSP patients carrying mutations in *SPG11*. Electron microscopy revealed that, in comparison to control lines, SPG11 neurons presented a large number of inclusions, membrane encircled structures and vacuoles of diverse electron density and size within neurites (Fig. 9A). The abundance of electron-dense vesicles and deposits might be indicative of alterations in the transport of membranous organelles in SPG11-dNeurons (20). This distinctive ultrastructural phenotype in SPG11 neurites, coupled with our mRNA expression analysis data, led us to investigate if alterations in SV transport were also present in SPG11-dNeurons. We thus performed time-lapse experiments on synaptophysin-mCherry^+^ hiPSC-dNeurons grown in microfluidic chambers (Fig. 9B). Our assays revealed that the transport activity in neurons derived from SPG11 patients were significantly different from controls (Fig. 9C and D). SPG11-dNeurons, in unison with our earlier hypothesis, presented significant reduction in anterograde transport of SV. Moreover, the number of axonal processes showing no SV transport and more retrograde transport was increased (Fig. 9D). Altogether, these data corroborated that the dysfunction of *SPG11* compromised the initiation of the SV movement and disturbed the critical balance of transport activity in SPG11 patients' neurons.

## DISCUSSION

To the best of our knowledge, this is the first report characterizing the importance of spatacsin for the homeostasis of cortical axons in human and mouse neurons. We detected a significant decrease of axon-related genes, neurite complexity and pathological accumulation of membranous bodies within neurites of SPG11-dNeurons. Our work underlined that spatacsin is preferentially expressed in human and mouse cortical neurons, is detected within neurites and growth cones and colocalizes with synaptic markers. To better elucidate the function, we knocked down spatacsin in mouse cortical neurons and showed axonal outgrowth defects, reductions in acetylated tubulin and deficiencies in anterograde axonal transport. Finally, acetyl-tubulin evaluation, and SV transport assays on SPG11-dNeurons pointed towards the dysfunction of *SPG11*, which leads profound deregulation of the axonal stability and transport. Taken together, this implies a crucial role of spatacsin in axonal functions.

### Spatacsin expression in human and mouse cortical neurons

The majority of our knowledge on *SPG11* is based on clinical observations in HSP patients. Our report is the first detailed characterization of spatacsin in hPSC-dNeurons. Our data show the preferential expression of spatacsin in human neurons compared with glia, in particular in human Ctip2^+^ cortical neurons. Such results were corroborated by our mouse cortical cultures and immunohistochemical analysis of mouse brain and were in line with previously published expression of spatacsin in non-diseased postmortem human cortical layer V (7).

In hPSC-dNeurons and mouse cortical neurons, spatacsin colocalized with SVs, microtubules and actin in axons and dendrites. In this regard, in synaptosomes, spatacsin signal overlapped with presynaptic and vesicle compartments. Further, biochemical analysis confirmed that spatacsin prominently colocalized with cytoskeletal markers in the cytosolic synaptosome fraction, suggesting a dynamic localization of spatacsin in synapses, possibly due to on and off cycling in SVs. Hirst and collaborators suggested that spatacsin, AP5 and spastizin form a coat-like complex where spatacsin localizes on the surface of the complex, functioning as protein scaffold, whereas spastizin would be the docker of the coat onto membranes and AP5 the protein sorter (6,8). Altogether, these results provide a comprehensive analysis of spatacsin expression in human and murine neural system, since previous reports in the spatacsin field were based on non-neuronal cell lines (6,7). More importantly, RT-PCRs on SPG11-dNeurons showing-specific downregulation of synaptic, motor and tubulin-associated markers, links the human expression to our observations in neuronal cultures and synaptosomes.

### Disruption of spatacsin leads to axonal pathologies in SPG11 patients

At present, there are 102 different mutations described in *SPG11* causative for AR-HSP; these comprise missense/nonsense (21), splicing (16), small indels (3), insertions (20) and deletions (22) (available at HGMD). The fact that HSP patients having different types of mutations in *SPG11* nevertheless present similar clinical phenotypes suggest that the majority of SPG11 cases lead to a loss of function of spatacsin (21). In this study, we describe for the first time axonal defects in hiPSC-dNeurons of patients with truncation and splice mutations in *SPG11.* The axonal defects in neurons from SPG11 patients match with results observed in spatacsin-silenced mouse cortical neurons, suggesting that spatacsin function is conserved in mammals. Our findings also indicate that loss of function of spatacsin may be causative for axonal dysfunction in SPG11 patients.

### Spatacsin function for axonal outgrowth

SPG11 patients’-derived neurons showed a significant reduction of the neurite complexity. A decreased neurite complexity present in neurons derived from SPG4 patients (11) suggests that neurite complexity defects may be a common pathology in different HSP subtypes. We also demonstrated a significant reduction in neurite length following spatacsin knockdown in mouse cortical neurons transfected before and after axon/dendrite specification. Significantly, shorter axons in siSPG11 both in early or late (Days 2 and 6) transfected neurons indicated that the reduction of spatacsin not only blocks axonal outgrowth but may also induce axonal retraction. This hypothesis is also supported by decrements in the anterograde transport rate and microtubule stability observed in SPG11-dNeurons and in spatacsin-silenced mouse neurons. The retraction of cortical axons induced by spatacsin dysfunction might be in accordance with the clinical findings of a TCC and the presumed retrograde degeneration of long cortical projections in SPG11 patients (4,5,22,23). Impaired cognition identified in SPG11 patients may be attributed to frontal lobe dysfunction and progressive frontal cortical atrophy (4,24). In addition, the reduction of transcallosal projections is associated in humans with deficiencies in abstract reasoning and problem solving (25,26). Thus, defects in the connectivity of the cortical neurons by the loss of function of spatacsin may explain the TCC and progressive decline associated with SPG11-induced AR-HSP. Interestingly, most of the neurons expressing spatacsin at high levels were located in the cortical Layers III and V, the topographical site of transcallosal and cortico-spinal projections, respectively (25,26).

### Dysfunction of spatacsin impairs cytoskeleton stability and axonal transport

The abundance of pleomorphic membranous material in suralis nerve biopsies of SPG11 patients is characteristic for a severe axonal neuropathy (4). The accumulation of vesicle-like bodies, the downregulation of motor genes and synaptic markers in SPG11-dNeurons as well as the impairment of SV transport in SPG11-dNeurons and spatacsin-silenced mouse axons, highly resemble this clinical observation and are possibly implying an alteration in the axonal transport and SV pathway. The SV cycle is mainly based on its initial excision from trans-Golgi network and its subsequent recycling in the early endosome (27). It was recently speculated that in other HSPs (e.g. *SPG47*, *SPG50*, *SPG51* and *SPG52)* the neuronal degeneration may be due to defects in the AP4 complex, which is associated with the transport between the trans-Golgi network and endosomes (9). This is interesting, since we observed a preferential impairment of the axonal traffic in the anterograde direction within axonal processes of SPG11-dNeurons and spatacsin-silenced mouse neurons.

It is well known that the acetylation of tubulin contributes to axonal connectivity by facilitating the stabilization of the microtubules and the recruitment of motor proteins to the tubulin rails (28,29). Interestingly, we observed that the dysfunction of spatacsin in human and mouse neurons induces a significant reduction of acetylated tubulin, which suggests that spatacsin may play an important role in the motor machinery by influencing tubulin-microtubule turnover. A previous report showed similar findings in a zebrafish model, wherein blockade of *spg11* expression presented outgrowth defects together with a marked reduction of acetylated tubulin (17).

By employing a complementary approach using human and mouse cortical neurons, we provide the first evidence that spatacsin has a major impact on neurite plasticity through maintaining cytoskeleton stability and SV transport. In the light of these results, we suggest that, due to transport and cargo trafficking deficiencies, the loss of function of spatacsin contributes to the profound failure of cortico-cortical and cortico-spinal projections observed in SPG11 patients. The analysis of hiPSC-derived neuronal cultures from SPG11 patients may reveal not only the significance of the loss of function in diseased human neurons but also define new targets to intervene the course of this progressing motor neurons disease.

## MATERIALS AND METHODS

### Patients

The patients included (*n* = 2; hereafter referred to as SPG11-1 and SPG11-2) were Caucasians with prototypical characteristics of AR-HSP, and genetically confirmed heterozygous mutations in both alleles of *SPG11*. SPG11-1 has heterozygous nonsense mutation at c.3036C > A/p.Tyr1012X in exon 16 and c.5798 delC/p.Ala1933ValfsX18 mutation in exon 30 (4,30). SPG11-2 has a heterozygous nonsense mutation at c.267G > A/p. Trp89X in exon 2 (4) and a splice site mutation 1457-2A > G in intron 6 [corresponding to the previously reported mutation c.1757-2A > G, (4)]. The controls (*n* = 2; hereafter referred to as Ctrl-1 and Ctrl-2) were healthy Caucasian individuals with no history of movement disorder or neurologic disease. The detailed clinical and genetic characteristics are summarized in Supplementary Material, Table S1. The human fibroblasts were obtained from dermal punch biopsies from one of the upper arm as previously described (11), following Institutional Review Board approval (Nr. 4120: *Generierung von humanen neuronalen Modellen bei neurodegenerativen Erkrankungen*) and informed consent at the movement disorder clinic at the Department of Molecular Neurology, Universitätsklinikum Erlangen (Erlangen, Germany). Fibroblasts were cultured in IMDM/Glutamax containing 15% fetal bovine serum (Invitrogen) and penicillin/streptomycin (Pen/Strep; Invitrogen).

### Generation of human neurons derived from hPSC

hiPSCs were generated from fibroblasts from SPG11 patients (SPG11-1 and SPG11-2) and control subjects (CTRL-1 and CTRL-2) as previously described (11). Briefly fibroblasts from patients and controls were reprogrammed by retroviral transduction using the Yamanaka factors, Kuppel-like factor 4, c-Myc, Oct4 (octamer-binding transcription factor 4) and SRY-box 2 (31).We established one hiPSC line from each patient (SPG11-11, SPG11-21) and Control (CTRL-12, CTRL-22). The generated hiPSC lines were characterized for the pluripotency markers Nanog and Tra1-60, and mutations in patient-derived hiPSC lines were reconfirmed. Finally, all the hiPSC lines were screened for stable karyotype using the G-banding chromosomal analysis (data not shown). All experiments with hESCs (HUES-6, Harvard University) were carried out in accordance with the German Stem Cell Act (RKI AZ. 1710-79-1-4-68). Both hiPSCs and hESCs, hereafter referred to as hPSCs, were cultured in 6-well plates (Corning) precoated with 0.5 mg Matrigel (BD Biosciences) and with mTeSR1 medium (Stemcell Technologies) under feeder-free conditions. For passaging, the hPSC clones were incubated with Collagenase IV (200 U/ml) for 20 min (min). The clones were randomly sliced into small chunks and later gently lifted off the plates using Costar cell lifter (Corning). To generate embryoid bodies (EBs), hPSC were transferred to ultra-low attachment plates (Corning). To induce neuronal differentiation, the EB colonies were maintained in suspension in neural induction medium (NIM: DMEM/F12/Glutamax supplemented with N2/B27 (Invitrogen) and Pen/Strep) for 1 week and then plated on polyornithine (PORN)/laminin (Invitrogen)-coated plates to obtain neural rosettes. Rosettes were formed within 1 week, manually dissected and cultured in neural proliferation medium [NPM: NIM supplemented with 20 ng/ml fibroblast growth factor 2 (Preprotech)] to form proliferative neural precursor cell (NPC) lines. We generated one NPC line from each patient (SPG11-111, SPG11-211) and Control (CTRL-122, CTRL-221). NPCs were maintained at high density, grown on PORN/laminin-coated plates in NPM and split ∼1:3 every 5–7 days with trypsin (TrypLE^TM^ Express; Invitrogen). Terminal differentiation of NPCs towards neuronal cells was initiated in neural differentiation medium [NDM: NIM supplemented with 20 ng/ml brain-derived neurotrophic factor (Preprotech), 20 ng/ml glial cell line-derived neurotrophic factor (Peprotech), 1 mm dibutyryl-cyclic AMP (Sigma–Aldrich) and 200 nm ascorbic acid (Sigma–Aldrich)] at a density of 40 000 cells/cm^2^ on PORN/laminin-coated plates or glass coverslips. Neuronal cultures were kept for differentiation under these conditions from 12 to 40 days with a half medium change every week. Since we have used one NPC/neuronal line from each control and SPG11 patient groups, for the sake of simplicity for the readers, we will hereafter refer to the neuronal lines as CTRL-1, CTRL-2, SPG11-1 and SPG11-2 from the control and SPG11 patients, respectively.

### Electron microscopy in hiPSC-dNeurons

Transmission electron microscopy in hPSC-dNeurons was performed as previously described (11,32).

### Evaluation of the neurite length and complexity in hiPSC-dNeurons

HPSC-dNeurons from SPG11 patients and controls were grown in microfluidic chambers (SND450, Xona Microfluidics, Temecula; Fig. 3C) as described before (11). The grooves in the microfluidic chambers enabled the axons to grow parallel and in unidirectional (Fig. 3C). A total of 60 000 NPCs were plated on the soma side and cells were cultured for 15 days in NDM. Axonal-like processes passing through the grooves were visualized using a Zeiss inverted fluorescent microscope (Zeiss). At least 20 cells per NPC line were imaged for analysis. Neurites of individual cells, starting from the end of the groove, were traced using NeuronJ (ImageJ; sbweb.nih.gov/ij/) to calculate neurite length and number of branching points.

### Real-time PCR

Total RNA was isolated from 4 weeks differentiated neurons of two controls (CTRL-1, CTRL-2) and two SPG11 patients (SPG11-1, SPG11-2) neuronal lines using RNAeasy mini kit (Qiagen). Five hundred nanograms of RNA were reverse transcribed to cDNA using QuantiTect RT-PCR kit (Qiagen) according to the manufacture instructions. RT-PCR was performed in 7300 Cycler Real Time PCR System (Applied Biosystems) using 1 µl of cDNA, transcript-specific primers (200 nm each) and 1× SybrGreen Master Mix (Applied Biosystems) in a total volume of 20 µl. The primer pairs used are listed in the Supplementary Material, Table S2.

### Animals

Wild-type C57BL/6 mice at embryonic (E15 and E18), postnatal (P10) and adult ages (P150) were used. All experiments were carried out in accordance with the European Communities Council Directive of 24 November 1986 (86/609/EEC).

### Primary murine cortical neurons

Cortices from mouse embryos at E15 were dissected, chopped into 200–500 µm tissue pieces, and mechanically dissociated with siliconated glass pipettes. Neurons were seeded at a range of 5 × 10^4^–5 × 10^6^ cells/cm^2^ on surfaces coated with poly-d-lysine (PDL; Sigma–Aldrich) and laminin (Invitrogen), and cultured in Neurobasal culture medium (Invitrogen) supplemented with l-glutamine, Pen/Strep and B27. Neurons were kept in culture from 1 to 21 days in 5% CO_2_, 90% humidity incubator at 37°C and harvested accordingly for immunofluorescence (IF) and immunoblotting (IB) procedures.

### Synaptosome preparations

Synaptosomes were prepared as previously published (33). Briefly, forebrains from three to four adult mice were dissected and kept in a cold glass-Teflon homogenizer together with 30 ml of prechilled 4 mm HEPES–NaOH; pH = 7.3 supplemented with 0.32 m sucrose. Tissue homogenization was performed by 10 up-down strokes at 900 rpm, and centrifuged at 800*g* for 10 min at 4°C. The resulting supernatant (S1) was centrifuged at 10 000*g* for 15 min at 4°C, and the newly obtained pellet (P2) at 10 000*g* for 20 min with the aim to isolate the synaptosome fraction (P3). Synaptosomes were broken by osmotic shock, and the synaptosomal plasmatic membranes (P4) separated by centrifugation at 25 000*g* for 20 min at 4°C. The resulting supernatant (S4) was further centrifuged at 30 000*g* overnight (ON) at 4°C in order to separate cytosolic fraction (S5) and SVs fraction (P5). Synaptosomal fractions were prepared for further IB assays. To perform IF on whole synaptosomes, the synaptosome pellet P2 was resuspended in 4 ml of 0.32 m sucrose, loaded on a discontinuous Ficoll gradient (4 ml of Ficoll at 12%, 1 ml at 9% and 4 ml at 5%) and centrifuged at 40 000*g* for 35 min at 4 °C. The synaptosomal-enriched fractions were collected at the two interfaces, between 5–9 and 9–12% of Ficoll concentration. After protein quantification, the synaptosomal fraction was distributed in aliquots of 0.5 mg and centrifuged at 20 800*g* for 12 min at 4°C and resuspended in 50 μl of sodium buffer (20 mm HEPES–NaOH, pH = 7.3; 10 mm glucose, 5 mm KCl, 140 mm NaCl, 5 mm NaHCO_3_, 1 mm MgCl_2_, 1.2 mm Na_2_HPO_4_). Then, synaptosomes were mounted on PDL-coated slides (Superfrost Plus, Thermo scientific). After fixation in 4% paraformaldehyde (PFA) and three washes with phosphate-buffered saline (PBS), mounted synaptosomes were incubated with blocking buffer (PBS-containing 0.2% gelatin and 20% of normal goat serum) for 60 min at room temperature (RT). Next, synaptosomes were incubated ON at 4°C with antibodies against spatacsin, the presynaptic marker SNAP25 (Synaptosomal-associated protein 25) or the SV marker VAMP2 in IF buffer 1 (IFB1: PBS-containing 0.2% gelatin and 1% normal goat serum). After three washes with PBS, synaptosomes were incubated for 60 min at RT with the fluorescent secondary antibodies diluted in IFB1, washed in PBS and finally mounted. Synaptosomes were examined using a Leica TCS-SL confocal microscope (CCiTUB, Biology Unit of Campus of Bellvitge, University of Barcelona, Spain).

### Acetyl-tubulin staining

HiPSC-dNeurons and GFP^+^ murine cortical neurons were fixed using ice-cold methanol at −20°C for 10 min followed by two rinses with PBS and a second fixation in 4% PFA at 37°C for 15 min (34,35). After three washes with PBS, neuronal cultures were stained with α-acetyl-tubulin (35) together with α-βIII-tubulin in hiPSC-dNeurons and with α-GFP in murine cortical neurons for further IF examinations employing Zeiss inverted fluorescent Apotome.2 and LSM-780 confocal microscope setups (Carl Zeiss).

### Measurement of neurite outgrowth and fluorescent signal in cortical neurons

Cotransfected (transfection protocols are indicated in Supplementary Material) murine cortical neurons were labeled with α-MAP2 (microtubule-associated protein 2) and α-TAU (microtubule-associated protein TAU) to differentiate dendrites and axons. Thereafter, axons and dendrites from transfected neurons were submitted to neurite length and branch-point quantifications using NeuronJ. Expression of acetylated tubulin and spatacsin in neurons was quantified *in situ* by measuring the fluorescent intensity quantitatively [(36) and ImageJ (http://rsbweb.nih.gov/ij/docs/guide/userguide-27.html)]. Fifty to 100 independent cells per experimental condition were analyzed.

### Synaptic vesicle transport experiments

Murine cortical neurons cultured for 4 days were transfected with either siLuc or siSPG11 along with pSynaptophysin-mCherry to visualize SV compartment and kept an additional 16–24 h in culture. Live-cell imaging was performed in synaptophysin-mCherry^+^ neurites in recording buffer 1 (10 mm HEPES, pH = 7.5; 120 mm NaCl, 3 mm KCl, 2 mm CaCl_2_, 2 mm MgCl_2_, 10 mm glucose; *Ω* = 240 mmol/kg), at stable temperature and balance CO_2_ conditions and employing an Nikon Eclipse Ti inverted fluorescent microscope (Nikon) equipped with a EMCCD camera (model DU-885, Andor Scientific Cameras) and NiS-Elements AR 3.2 OS (Nikon). Time-lapse recordings were designed as follows: neuronal cell bodies were included in the camera field to measure anterograde and retrograde transport (Fig. 7A). Recording conditions were setup as 1 frame/s with a total duration time of 10 min. At least 20 neurites per experimental condition were recorded and analyzed. To obtain SV transport data, time-lapse videos were converted into 2D kymographs by mathematical algorithms (ImageJ).

Neurons derived from controls (CTRL-1 and CTRL-2) and SPG11 (SPG11-1 and SPG11-2) patients' iPSC were grown on microfluidic chambers, 80 × 10^3^ per chamber in coculture with 1 × 10^4^ human primary astrocytes (ScienceCell Research Laboratories) in the soma side and with 2 × 10^4^ human astrocytes plated into the axonal side as published previously (11). After 2 weeks in culture, hiPSC-dNeurons were then infected with lentivirus (LV) encapsulating a synaptophysin-mCherry construct (see details in Supplementary Material) (33). Six days after LV infection, live-cell imaging was performed in synaptophysin-mCherry^+^ axonal processes passing through the grooves (Fig. 9B) in recording buffer 2 (10 mm HEPES, pH = 7.5; 144 mm NaCl, 2.5 mm KCl, 2.5 mm CaCl_2_, 2.5 mm MgCl_2_, 10 mm glucose; *Ω* = 309 mmol/Kg) at stable temperature and balance CO_2_ conditions, and employing an Nikon Eclipse Ti inverted fluorescent microscope (Nikon) equipped with a EMCCD camera. Recording conditions were setup as 1 frame/s with a total duration time of 10 min. At least 20 neurites per experimental condition were recorded and analyzed. To obtain SV transport data, time-lapse videos were converted into 2D kymographs by mathematical algorithms (ImageJ). For each evaluated hPSC-dNeuron line, axonal processes were classified as they had SVs in motion; and their net transport direction (anterograde and retrograde) was calculated by subtracting the average distance per SV moving anterogradely and retrogradely [(anterograde Σd*x*/anterograde SVs) − (retrograde Σd*x*/retrograde SVs)].

### Statistical analysis

Immunofluorescent signals, synapse counting, neurite measurements and SV transport data were statistically analyzed using Prism software (GraphPad). The data were shown as mean ± SD. Statistical analysis was carried out employing the Student's *t*-test for unpaired variables (two-tailed), one- or two-way ANOVA followed by Bonferroni multiple comparison tests when three or more groups were compared. *P*-values of <0.05 were considered significant.

## SUPPLEMENTARY MATERIAL

Supplementary Material is available at *HMG* online.

## FUNDING

Support for this study came from the Tom-Wahlig Foundation Advanced Fellowship and the Interdisciplinary Centre for Clinical Research (IZKF, University Hospital of Erlangen). Additional support came from the German Federal Ministry of Education and Research (BMBF, 01GQ113), the Bavarian Ministry of Education and Culture, Science and the Arts in the framework of the Bavarian Molecular Biosystems Research Network and ForIPS and the German Research Society (Deutsche Forschungsgemeinschaft) Grant No. INST 410/45-1 FUGG. Funding to Pay the Open Access Charge was provided by the German Federal Ministry of Education and Research (BMBF, 01GQ113).

## Supplementary Material

Supplementary Data
