# Supplementary material for: Dysfunction of spatacsin leads to axonal pathology in SPG11-linked hereditary spastic paraplegia
Source: Hum Mol Genet. 2014 May 2;23(18):4859–74. doi: 10.1093/hmg/ddu200 (PMC4140466; doi:10.1093/hmg/ddu200)
Supplement: Supplementary Data [file supp_23_18_4859__index.html]

Dysfunction of spatacsin leads to axonal pathology in SPG11-linked hereditary spastic paraplegia — Dysfunction of spatacsin leads to axonal pathology in SPG11-linked hereditary spastic paraplegia — Supplementary Data 

# Dysfunction of spatacsin leads to axonal pathology in *SPG11*-linked hereditary spastic paraplegia

## Supplementary Data

Supplementary Data

**Files in this Data Supplement:**

- Supplementary Data - Docx file
- Supplementary Figures - doc file
- Supplementary Table 1 - docx file
- Supplementary Table 2 - docx file
- Supplementary Video 1 - mov file
- Supplementary Video 2 - mov file
