# Supplementary material for: Dysfunction of spatacsin leads to axonal pathology in SPG11-linked hereditary spastic paraplegia
Source: Hum Mol Genet. 2014 May 2;23(18):4859–74. doi: 10.1093/hmg/ddu200 (PMC4140466; doi:10.1093/hmg/ddu200)
Supplement: Supplementary Data [file supp_ddu200_ddu200supp.docx]

**SUPPLEMENTARY INFORMATION**

**Antibodies and Reagents**

Polyclonal antibodies against spatacsin (mouse and rabbit; 1:250 each) and spastizin (rabbit; 1:250) were supplied by Proteogenix. The axonal marker α-TAU (microtubule-associated protein TAU; goat; 1:250) was purchased from Santa Cruz, and the dendritic markerα-MAP2 (microtubule-associated protein 2; mouse; 1:200) from Sigma Aldrich. The following antibodies α-MARCKS (myristoylated alanine-rich C-kinase substrate; rabbit; 1:200), α-Ctip2 (Coup TF interacting protein 2; rat; 1:300), α-GFAP (glial fibrillary acidic protein;goat; 1:1000) and α-PSD95 (postsynaptic density protein 95; goat; 1:200) were obtained from Abcam, whereasα-vGlut2 (vesicle glutamate transporter 2; rabbit; 1:1000),α-vGAT (vesicle GABA transporter; rabbit; 1:250), α-VAMP2 (vesicle-associated membrane protein 2; mouse and rabbit; 1:2000 each) and α-SNAP25 (synaptosomal-associated protein 25; clone SMI-81; mouse; 1:2000) from Synaptic Systems (SySy). The neuronal markers α-calbindin (rabbit; 1:250) was purchased from Cell Signaling, α-NeuN (clone A60; mouse; 1:250) from Millipore and α-βIIItubulin (clone TUJ1; rabbit and mouse; 1:2000 each) from Covance. The synaptic markers α-synaptophysin (clone p38; mouse; 1:1000), α-syntaxin1 (clone HPC-1; mouse; 1:2000) and the cytoskeletal markersα-acetyl-tubulin (mouse; 1:2000), α-βactin (mouse; 1:1000) were obtained from Sigma Aldrich. Additionally, the antibodiesαγadaptin (BD Transduction Labs; mouse; 1:2000) and α-GADPH (glyceraldehyde 3-phosphate dehydrogenase; mouse; 1:4000; Calbiochem) andα-GFP (green fluorescent protein; rabbit; 1:2000; Invitrogen) were used as loading control markers. In addition,α-Oct4 (octamer-binding transcription factor 4; clone c-10; mouse; 1:200) was purchased from Santa Cruz, and α-alpha-synuclein (clone LB509; mouse; 1:500) from Covance.

Phalloidin-Alexa566 (1:500; Invitrogen) was acquired from Invitrogen. The siRNA sequence used to knock down SPG11 (siSPG11) was previously reported ([Murmu *et al.*, 2011](#_ENREF_21)) and purchased from Santa Cruz. SiRNA to knock down fly luciferase (siLuciferase; siLuc) was obtained from Shanghai GenePharma. Expression vectors pEGFP (Clontech), pEGFP-hSpatacsin, pEF1-dTomato, pEZX-SPG11-GFP (GeneCopoeia), and pSynaptophysin-mCherry were used for either transfections or cotransfections together with siRNA.

**Cloning of the full sequence of human spatacsin**

The oligonucleotides 5’-GCTGCAGAGGAAGGGGTCGC-3’ and5’-ACCTGCTAGCATGTCCTTTAG-3’ were employed as forward and backward primers respectively to amplify the full sequence of spatacsin from human cDNA,Spatacsin amplicon was subsequently cloned into the mammal expression vector pEGFPC2. Recombinant GFP-spatacsin (GFP-Spat) was detected with α-GFP and α-spatacsin antibodies in IB from transfected cells.

**Culture preparation of human cell lines and astrocytes**

Human neuroblastoma cell line (SH-SY5Y), and human embryonic kidney 293 cells (HEK293) were cultured according producer’s instructions. Human primary cerebellar astrocytes (HA-c) were obtained from ScienceCell (#1810) and cultured according to producers’ instructions. SH-SY5Y, HA-c, and transfected HEK293 were harvested for further IB assays.

**Immunofluorescence (IF)**

Cultures of mouse cortical neurons and hPSC-dNeurons were fixed in 4 % PFA for 15 min at RT. After several rinses with PBS, cultures were pre-incubated for 60 min at RT with PBS supplemented with 0.1 % Triton x-100 (PBST) and incubated ON at 4 °C with primary antibodies diluted in immunofluorescence buffer 2 (IFB2: PBST supplemented with 5 % normal donkey serum). Next, samples were rinsed several times with PBST, and incubated with suitable fluorescent secondary antibodies diluted in IFB2 for 60 min at RT. Moreover, F-actin was detected by incubating fluorescent phalloidin diluted in PBS for 30 min at RT. After several washes with PBS, cells were mounted for further microscopic analysis. All IFs were visualized using a Zeiss inverted fluorescent Apotome.2 and LSM-780 microscopesetups (Carl Zeiss).

Adult mice were transcardiallyperfused with PBS and 4% PFA; and the brains were dissected, and coronally sliced at 35 µm thickness using a Leica SM-2010R cryostat (Leica). Brain sections thus were submitted to epitope retrieval in citrate buffer (DAKO) for 30 min at 80 ^o^C, rinsed several times in TBS^+^ (Tris-buffered saline and 0.05 % Triton x-100) at RT, blocked with blocking solution (TBS^+^ supplemented with 3 % normal donkey serum and 3 % Triton x-100), and incubated with primary antibodies ON at 4 ^o^C. After several rinses, sections were incubated with suitable fluorescent secondary for 1 at RT, rinsed again in Tris-buffered saline (TBS), mounted for further examination in a LSM-780 microscopesetup (Carl Zeiss).

**Protein sample preparation**

Protein samples from brain tissue, synaptosomal fractions, cell lines, mouse cortical neurons, and hPSC-dNeurons were prepared using cold homogenization buffer (50 mM Tris, pH = 8.0; 2 mM EDTA, 140 mM NaCl) supplemented with 1 mM dithiothreitol (DTT) as well as protease and phosphatase inhibitors. After homogenization, samples were centrifuged first at 800 ×*g* for 10 min to remove nuclear fraction. Thus, supernatants (S1) were incubated with a final concentration of 1% of Triton x-100 for 30 min on ice and centrifuged at 11,000 ×*g* for 10 min at 4ºC. Resultant samples were submitted to protein evaluation and IB assays.

**Immunoblotting (IB)**

5 to 40 μg of total protein were loaded on 4 – 12 % gradient gels (Invitrogen) for further SDS-PAGE separation, and blotted onto nitrocellulose membranes. Proper primary antibodies were incubated overnight at 4 ºC in Tris-buffered saline and 0.1 % Tween-20 (TBST) supplemented with 5% non-fatty dry milk. After several washes with TBST, blots were incubated with corresponding secondary antibodies conjugated to horseradish peroxidase and developed by chemical luminescence techniques. Each experiment was replicated at least 3 times.

**Transfection methods, and Lentiviral infection**

HEK293, primary mouse cortical neurons, and hPSC-dNeurons cultures were transfected using Lipofectamine 2000 (Invitrogen) and Mouse Nucleofection (Lonza) according to manufacturer instructions. 30 - 40 pmol siRNA per 1 μg DNA were employed in cotransfections.

hiPSC-dNeurons already grown on microfluidic chambers for two weeks were infected with lentivirus (LV) encapsulating mCherry-synaptophysin construct in a multiplicity of infection (MOI) equal to 2. Media was replaced 48 hours after LV infection, and hPSC-dNeuron cultures were kept in culture for 5-6 days more to ensure the proper visualization of mCherry-synaptophysin within the axonal processes.

**Supplementary Video 1**: siSPG11^+^ neurite. Arrow (magenta) indicates anterograde transport direction.

**Supplementary Video 2:** siLuc^+^ neurite. Arrow (magenta) indicates anterograde transport direction.
