# Supplementary material for: Dysfunction of spatacsin leads to axonal pathology in SPG11-linked hereditary spastic paraplegia
Source: Hum Mol Genet. 2014 May 2;23(18):4859–74. doi: 10.1093/hmg/ddu200 (PMC4140466; doi:10.1093/hmg/ddu200)

Supplementary Figure 1 Spatacsin antibody specifically recognizes spatacsin full length. (A) IB of embryonic brain (Brain E18) and cortical neurons (C. Neurons) probed with either spatacsin antibody (α-spatacsin blot) or Ig kappa isotype control (Isotype Blot). ßIIItubulin was used as loading control. (B) Blots of Non-transfected HEK293 (MOCK) and transfected either with pEGFP expression mammal vector (pGFP) or with the GFP-tagged human spatacsin (pGFP-Spat) showed the expression of the GFP-tagged human spatacsin at expected size of 300 KDa by probing with the antibodies α-GFP and α-spatacsin. GADPH marker was used as loading controls. (C) Blots of non-transfected HEK293 (MOCK) and transfected with the siLuc or siSPG11 were probed with α-spatacsin antibody. βactin was used as loading control.

Supplementary Figure 2 SPG11 is expressed in murine cortical neurons. (A) Scheme illustrating mouse cortical cultures transfected with either CMV::GFP (pCMV-GFP) or SPG11::GFP (pSPG11-GFP) vectors. Cultures transfected with CMV::GFP (CMV-GFP) showed GFP expression in all types of cells, whereas after transfection with SPG11::GFP (SPG11-GFP), the GFP signal was almost exclusively detected in neurons. (B) Mixed mouse cortical cultures were transfected with either CMV::GFP (CMV-GFP) or SPG11::GFP (SPG11-GFP) vectors. Neurons were labeled with α-MAP2. Yellow arrows indicated GFP+ neurons whereas cyan arrowheads GFP+ non-neuronal cells; scale bar = 50 µm. (C) Graph representing the percentage of different types of cells expressing GFP in mixed cultures transfected with CMV::GFP (CMV-GFP) and SPG11::GFP (SPG11-GFP) respectively. Note that more than 97 % of the SPG11::GFP transfected cells are neurons. (D) Mouse cortical cultures were transfected with the SPG11::GFP (SPG11-GFP) vector and probed with α-vGlut2, α-vGAT and α-GFAP to label projecting neurons, interneurons, and astrocytes. Cortical neurons, but not astrocytes were able to express GFP (arrows). Scale bar = 50 µm.

Supplementary Figure 3 Spatial characterization of spatacsin expression in mouse brain. (A) Blots of different mouse brain regions showing expression of spatacsin at embryonic (E18) and adult ages (P150). GADPH was used as loading control. Cortex (Ctx), cerebellum (CB), hippocampus (HC), thalamus (TH), spinal cord (SC), and SH-SY5Y cells were examined. (B) Panoramic overview of the cortical column probed with α-spatacsin, α-NeuN as neuronal marker and α-GFAP as glial marker. Spatacsin+ cells were also posive for the neuronal marker NeuN (arrowheads). Cortical layers (from I to VI) and white matter (WM) were indicated accordingly. Scale bar = 100 µm. (C) Detailed micrograph of the cortical layer V confirmed that NeuN+ neurons (red) express spatacsin (green) at low (arrowheads), and at high levels (arrows and inset). Scale bar = 20 µm. (D) Micrographs revealed that NeuN+ (red and arrowheads) but no GFAP+ cells (magenta and arrows) express spatacsin (insets). Scale bar = 20 µm.

Supplementary Figure 4 Spatacsin antibody detects spatacsin in postmitotic neurons. (A - C) Cortical neurons labeled with ßIIItubulin (red) were coincubated with Ig kappa isotype control (Isotype; green) to prove the specificity of the signal (D - F) α-spatacsin antibody previously pre-incubated with spatacsin peptide (green) was incubated together with ßIIItubulin (red). (G - I) α-spatacsin antibody (green) was incubated together with ßIIItubulin (red). Scale bar = 20 µm.

Supplementary Figure 5 Transfection of siSPG11 in mouse cortical neurons promotes spatacsin knockdown. Mouse cortical neurons transfected with GFP together with siLuc or siSPG11 (green) were probed with α-spatacsin antibody (red). Scale bar = 5 µm.


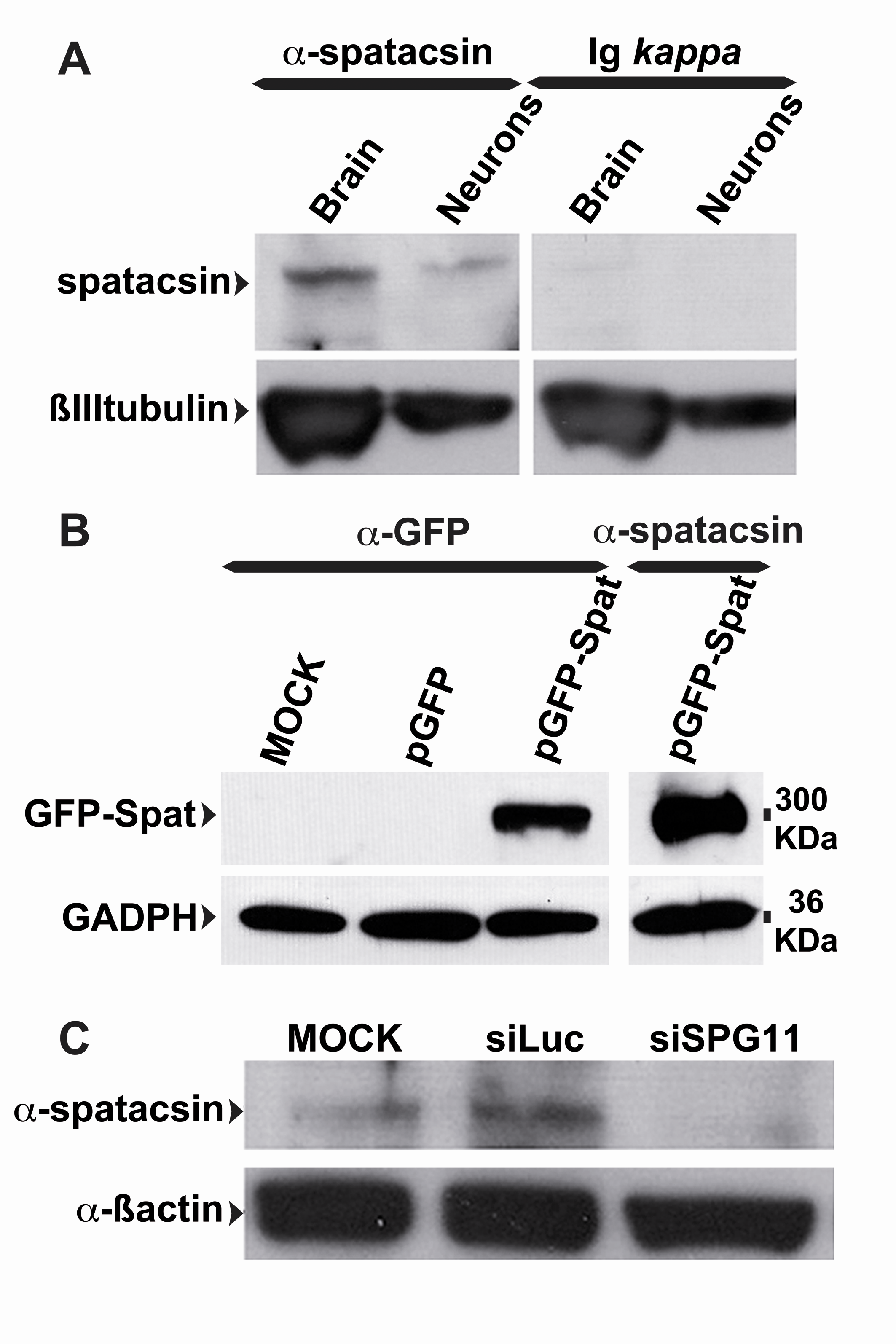


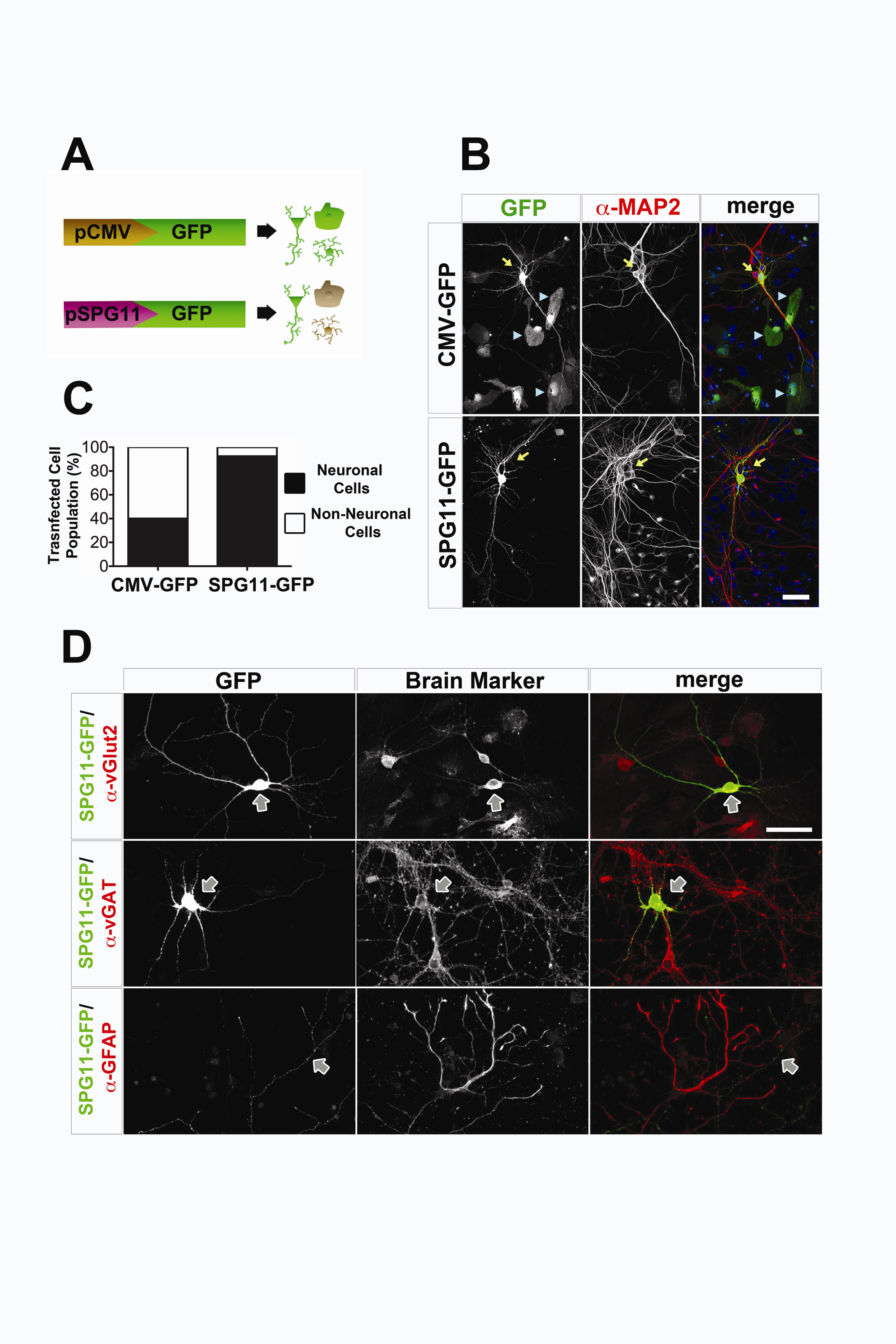


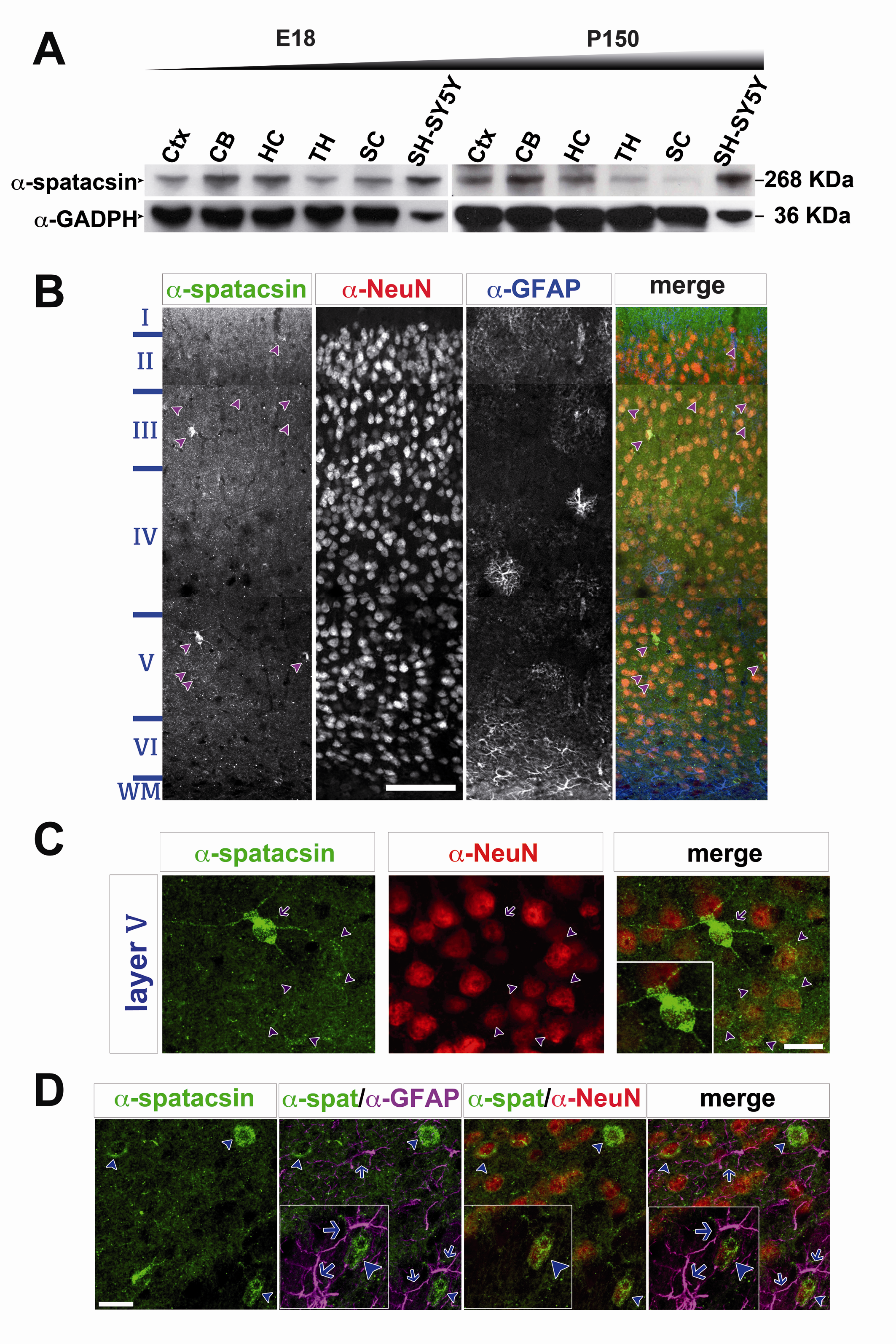


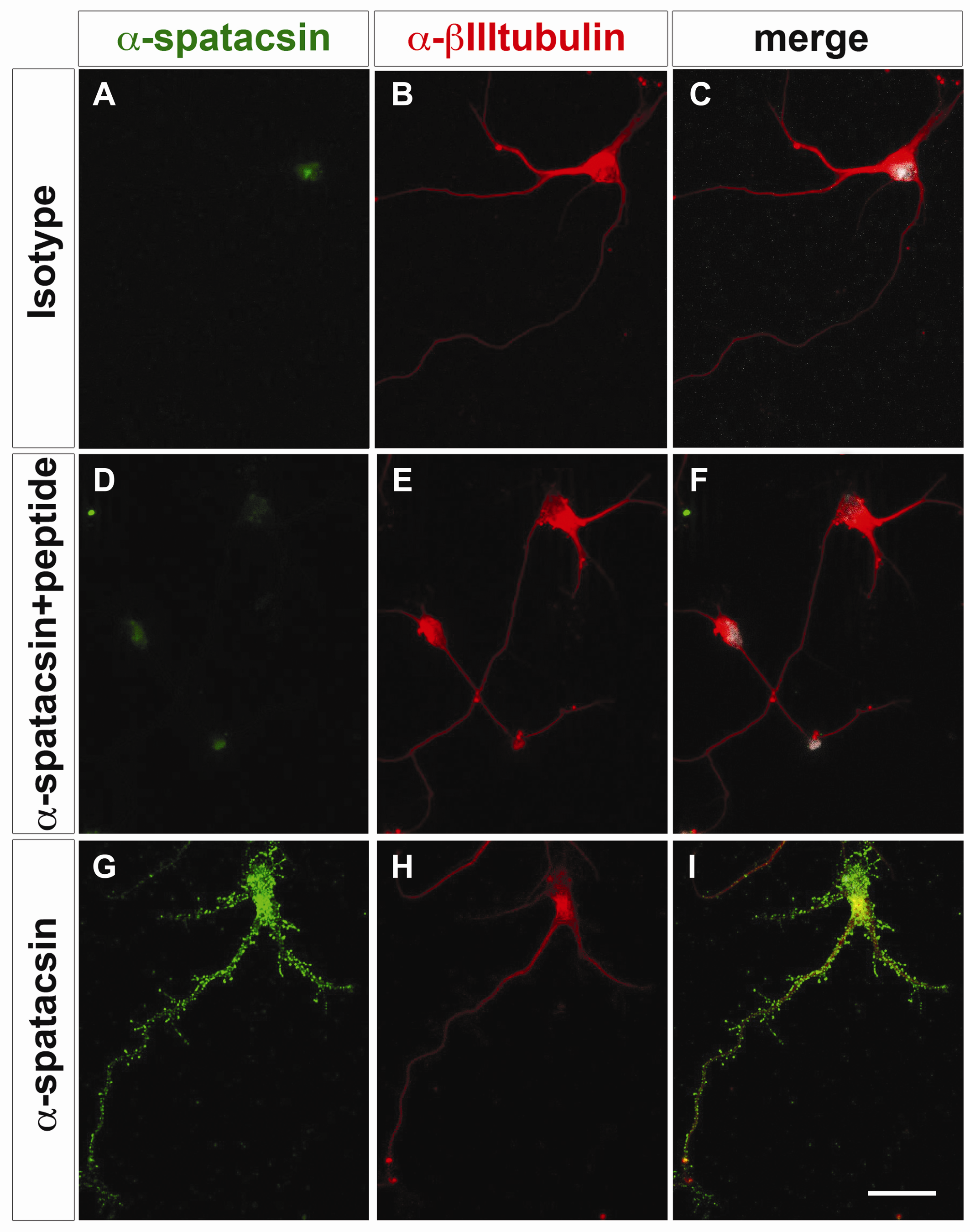


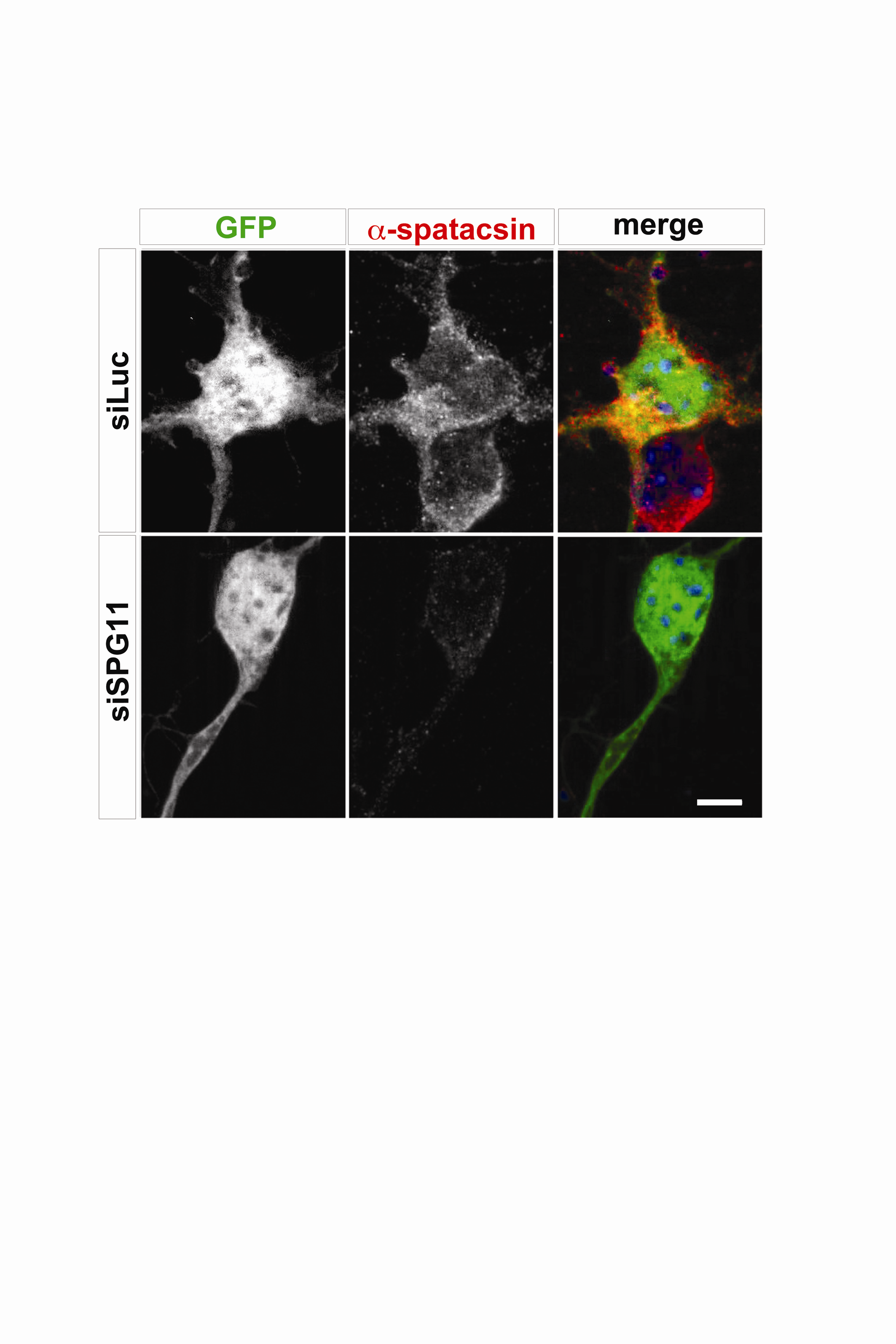

Supplement: Supplementary Data [file supp_ddu200_ddu200supp_figs.doc]
