# Supplementary material for: Dysfunction of spatacsin leads to axonal pathology in SPG11-linked hereditary spastic paraplegia
Source: Hum Mol Genet. 2014 May 2;23(18):4859–74. doi: 10.1093/hmg/ddu200 (PMC4140466; doi:10.1093/hmg/ddu200)
Supplement: Supplementary Data [file supp_ddu200_ddu200supp_table1.docx]

|  | Mutation | Sex | HSPRS  (max.52) | 3Tesla MRI | Age at onset  / Examination  (Y) | Landmark of disability | Barthel-Index | Cognitive impairment | Muscle wasting (upper / lower limbs) | Motor-sensory neuropathy |
| --- | --- | --- | --- | --- | --- | --- | --- | --- | --- | --- |
| SPG11-1 | Exon 16:  c.3036C > A heterozygote p.Tyr1012X  Exon 30:  c.5798 del C  heterozygot  p.Ala1933ValfsX18 | F | 39 | TCC, WML, cortical atrophy | 25 / 40 | 4 | 30 % | + | + | + |
| SPG11-2 | Exon 2:  c.267G > A  p. Trp89X  Intron 6:  1457-2 A > G  splice mutation | F | 35 | TCC, WML, cortical atrophy | 31 / 44 | 4 | 55 % | + | + | + |
| CTRL-1 | - | F | - | - | - / 53 | - | 100 % | - | - | - |
| CTRL-2 | - | F | - | - | - / 46 | - | 100 % | - | - | - |

Table 1: Clinic of SPG11 patients and control subjects.

Patients: SPG11-1, SPG11-2, controls: CTRL-1, CTRL-2, F: Female, HSPRS: hereditary spastic paraplegia rating scale, MRI: magnetic resonance imaging, TCC: thin corpus callosum, WML: white matter lesion, Y: years, Barthel index of activity of daily living (max. 100%).
