# Supplementary material for: Dysfunction of spatacsin leads to axonal pathology in SPG11-linked hereditary spastic paraplegia
Source: Hum Mol Genet. 2014 May 2;23(18):4859–74. doi: 10.1093/hmg/ddu200 (PMC4140466; doi:10.1093/hmg/ddu200)
Supplement: Supplementary Data [file supp_ddu200_ddu200supp_table2.docx]

Table 2: Primers used for qRT-PCR analysis

| Genes | Forward Primer | Reverse Primer |
| --- | --- | --- |
| *KIF5A* | TTACCTGGACAAAATTCGTGACC | GGTGACAGCCACATGACGAT |
| *KIF3A* | CTGCCTTGGTTGATGGAAAAAG | CGATTGGCATACCGTAATGTACT |
| *KLC1* | CAGCTAACCTACTGAATGATGCC | GCTCTTTTACACAACGGCTCT |
| *DYNC1LI2* | GGCTAGTGTTTTACGTGAGCA | TGGGGAACCTTGACAACCTTC |
| *PSD95* | GTGGAGGAGATTCGAGGCTTC | ACGAACTTTGTTTGCTGTCTTCT |
| *VAMP2* | CTCAAGCGCAAATACTGGTGG | TGATGGCGCAAATCACTCCC |
| *TTBK1* | GCTGTGGCAGGAACGAGAA | AGTTTGAAGGCTTGATGTCACG |
| *MAPT* | GTGGCCAGGTGGAAGTAAAATCT | GGTCAGCTTGTGGGTTTCAATCT |
| *SYN1* | AGCTCAACAAATCCCAGTCTCT | CGGATGGTCTCAGCTTTCAC |
| *SYT12* | CAAAGGCAGTCTCAGCATTGA | CCAAAGGTGTTGCTCACGG |
